# What are the sources of contraceptives for married and unmarried adolescents: Health services or friends? Analysis of 59 low- and middle-income countries

**DOI:** 10.3389/fpubh.2023.1100129

**Published:** 2023-02-06

**Authors:** Franciele Hellwig, Aluísio J. D. Barros

**Affiliations:** ^1^International Center for Equity in Health, Federal University of Pelotas, Pelotas, Brazil; ^2^Postgraduate Program in Epidemiology, Federal University of Pelotas, Pelotas, Brazil

**Keywords:** family planning, source of method, private sector, public sector, contraceptives, low-and middle-income countries, national health surveys

## Abstract

**Background:**

Despite the efforts to promote universal coverage for family planning, inequalities are still high in several countries. Our aim was to identify which sources of contraceptives women mostly rely on in low- and middle-income countries (LMICs). We also explored the different sources according to age and marital status.

**Methods:**

We used data from national health surveys carried out in 59 LMICs since 2010. Among all sexually active women at reproductive age, we explored inequalities in demand for family planning satisfied by modern methods (mDFPS) and in the source of modern contraceptives according to women's age, classified as: 15–19, 20–34, or 35–49 years of age. Among adolescents, mDFPS and source of method were explored by marital status, classified as married or in union and not married nor in a union.

**Results:**

mDFPS was lower among adolescents than among adult women in 28 of the 59 countries. The lowest levels of mDFPS among adolescents were identified in Albania (6.1%) and Chad (8.2%). According to adolescents' marital status, the pattern of inequalities in mDFPS varied widely between regions, with married and unmarried adolescents showing similar levels of coverage in Latin America and the Caribbean, higher coverage among unmarried adolescents in Africa, and lower coverage among unmarried adolescents in Asia. Public and private health services were the main sources, with a lower share of the public sector among adolescents in almost all countries. The proportion of adolescents who obtained their contraceptives in the public sector was lower among unmarried girls than married ones in 31 of the 38 countries with data. Friends or relatives were a more significant source of contraceptives among unmarried compared to married adolescents in all regions.

**Conclusions:**

Our findings indicate lower levels of mDFPS and lower use of the public sector by adolescents, especially unmarried girls. More attention is needed to provide high-quality and affordable family planning services for adolescents, especially for those who are not married.

## 1. Introduction

Over the past decades, most of the low- and middle-income countries (LMICs) presented an increase in their levels of demand for family planning satisfied with modern methods (mDFPS) (1–4). Along with these improvements in utilization, more emphasis has been directed to the quality of services (5, 6). However, large inequalities within countries are still being reported in terms of wealth, area of residence, women's education, and especially between adolescents and older women (2, 4, 7–10).

To satisfy the women's demand for family planning, it is essential to offer effective and respectful care, providing comprehensive family planning information and a wide choice of methods (6). A key aspect of universal health coverage is equitable access to high-quality services without discrimination or undue financial hardship. Although some countries have based their strategies to increase modern contraceptive use on the public sector, several others have argued that it would be necessary to involve private and non-governmental organizations to achieve universal access (5, 11, 12). The chosen approach for family planning supply influences more than only the cost of the services. Public and private facilities vary on a range of characteristics that may influence women's decisions on whether to use a contraceptive method and if so, which. Other relevant characteristics are the geographical access of the health service, its reputation, level of privacy, provision of knowledge about family planning, and its suitability to meet the needs of specific subgroups (5, 13–15). These characteristics are highly variable between public and private facilities, which is especially important for unmarried adolescents who are more often underserved by family planning policies and subject to unfavorable attitudes by health providers and community leaders in several countries (9, 15–19).

The literature investigating patterns of family planning provision in low- and middle-income countries largely presents average estimates by world regions, indicating that the private sector is the main source of short-acting methods which are usually preferred by younger, wealthier, more educated, and urban women (12, 20). On the other hand, the public sector tends to be the main source of long-acting and permanent methods (12, 20). Fewer studies presented results at country level. Most of them investigated only one country or a limited number of countries. Comparing their findings, a huge variability across countries is observed (19, 20).

In this article, we used survey data from LMICs covering all world regions to investigate within- and between-country inequalities in levels of mDFPS and in the source of the contraceptives among adolescents. We compared adolescents with older women at the start to set the stage for the analyses. Next, we explored differences according to adolescents' marital status.

## 2. Materials and methods

We used publicly available data from the Demographic and Health Surveys (DHS), which are nationally representative, cross-sectional household surveys conducted in LMICs. We selected the most recent survey for each country, carried out since 2010 that collected information on family planning and method source, with 59 surveys included in the analyses. The surveys included information on all sexually active women, except for Afghanistan, Bangladesh, Pakistan, Turkey, Egypt, Jordan, and Yemen, where information was collected only for ever-married women. Women were considered sexually active if they were married or living with a partner, or if they reported having had sexual intercourse in the month preceding the interview.

### 2.1. Demand for family planning satisfied and sector of provision

Our main outcome is demand for family planning satisfied with modern methods (mDFPS), defined as the proportion of sexually active women in need of contraception who were using (or whose partners were using) a modern contraceptive method. A woman was considered in need of contraception if she was fecund and did not want to become pregnant within the next 2 years, or if she was unsure about whether or when she wanted to become pregnant. Pregnant women with a mistimed or unintended pregnancy were also considered in need of contraception. Methods were classified as modern if they were medical procedures or technological products (21), including oral contraceptive pills, injections, male and female condoms, diaphragms, spermicidal agents, emergency contraception, intrauterine devices (IUD), implants, and sterilization (female or male).

Current contraceptive users were asked where they last obtained their contraceptive method. We classified it into five groups: (I) public, including all governmental medical facilities, public community health workers, public pharmacies, and government distributions; (II) private, considering private hospitals, clinics, doctors, pharmacies, drug stores, market/shops, and vending machines; (III) non-profit, including facilities of non-governmental organization and faith-based facilities; (IV) friends or relatives; and (V) other sources (missing or unclassified source). Mixed facilities were classified as public providers, following the DHS definition.

### 2.2. Stratifiers

mDFPS and source of method were explored considering women's age, classified into three groups: 15–19 years (adolescents), 20–34 years, and 35–49 years. Adolescents were further classified as currently married (or in a union) or not.

### 2.3. Statistical analyses

The proportions of women with mDFPS by age group and by method source were calculated taking into account the complex survey design, including sample weights, clusters, and strata. Countries were grouped according to UNICEF world regions (Eastern and Southern Africa, West and Central Africa, Middle East and North Africa, Eastern Europe and Central Asia, South Asia, East Asia and the Pacific, Latin America and the Caribbean).

The percentages of mDFPS with 95% confidence intervals were presented in bar graphs while 95% confidence intervals of the estimates on the share of the source of method by women's age and adolescents' marital status were presented in the Supplementary material.

All analyses were performed using Stata (StataCorp. 2021. Stata Statistical Software: Release 17. College Station, TX: StataCorp LLC.) using publicly available anonymized databases. Institutions and national agencies that were responsible for the data collection in each country obtained ethics approval for the surveys.

## 3. Results

Our study sample included data from 59 LMICs comprising 784,996 sexually active women of reproductive age of which 59,531 were adolescents. The proportions of countries studied in each world region were 76% in West and Central African countries, 71% in Eastern and Southern Africa, 20% in Middle East and North Africa, 26% in Eastern Europe and Central Asia, 50% in South Asia, 40% in East Asia and Pacific, and 22% in Latin America and the Caribbean. mDFPS ranged from 6.1% in Albania to 85.6% in Colombia. The percentage of sexually active adolescents in each country varied from 0.9 to 33.7% of all sexually active women (Table 1).

**Table 1:** Sample size and demand for family planning satisfied by modern methods (mDFPS) among women of reproductive age from 59 low- and middle-income countries.

**Country**	**Sample (unweighted)**	**Percentage of adolescents**	**mDFPS (95% CI)**
**West and Central Africa**			
Benin (2017)	6,055	8.8	25.3 (23.9; 26.8)
Burkina Faso (2010)	5,800	8.5	38.1 (36.3; 40.0)
Cameroon (2018)	4,479	12.7	38.2 (36.1; 40.3)
Chad (2014)	3,854	12.3	14.7 (13.1; 16.4)
Congo Brazzaville (2011)	5,335	13.9	32.9 (30.9; 35.0)
Congo DR (2013)	6,685	11.6	16.2 (14.5; 17.9)
Côte d'Ivoire (2011)	3,755	12.2	28.2 (26.1; 30.4)
Gabon (2012)	3,696	14.9	39.6 (36.6; 42.8)
Gambia (2019)	3,456	8.3	39.5 (37.1; 41.9)
Ghana (2014)	3,561	5.5	37.7 (35.2; 40.2)
Guinea (2018)	2,916	11.1	24.9 (22.0; 28.0)
Liberia (2019)	3,896	15.2	44.6 (41.5; 47.7)
Mali (2018)	3,414	12.2	39.6 (36.9; 42.2)
Mauritania (2019)	4,498	9.7	27.7 (25.8; 29.6)
Niger (2021)	2,041	12.9	41.2 (37.0; 45.4)
Nigeria (2018)	11,538	7.4	30.6 (29.3; 31.9)
Senegal (2019)	2,811	7.9	52.1 (49.2; 55.1)
Sierra Leone (2019)	6,087	12.4	49.1 (47.3; 51.0)
Togo (2013)	3,863	6.9	34.1 (32.0; 36.2)
**Eastern and Southern Africa**			
Angola (2015)	4,953	15.5	26.8 (23.9; 29.9)
Burundi (2016)	5,688	2.5	38.5 (36.7; 40.3)
Comoros (2012)	1,807	7.6	26.2 (23.5; 29.1)
Ethiopia (2016)	5,053	7.1	60.4 (57.3; 63.3)
Kenya (2014)	6,915	4.5	70.2 (68.7; 71.7)
Lesotho (2014)	3,207	9.1	76.3 (74.3; 78.2)
Malawi (2015)	13,140	9.2	73.2 (72.1; 74.2)
Mozambique (2015)	4,422	33.7	50.3 (47.6; 53.0)
Namibia (2013)	3,761	9.3	78.1 (76.4; 79.8)
Rwanda (2019)	6,009	1.6	71.9 (70.5; 73.2)
South Africa (2016)	3,557	8.6	75.7 (73.7; 77.5)
Tanzania (2015)	5,437	9.6	52.9 (50.8; 54.9)
Uganda (2016)	8,283	9.6	50.3 (48.8; 51.9)
Zambia (2018)	5,926	9.2	65.0 (63.3; 66.7)
Zimbabwe (2015)	5,019	7.2	84.4 (82.9; 85.7)
**Middle East and North Africa**			
Egypt (2014)	14,288	3.5	80.0 (79; 80.9)
Jordan (2017)	8,882	3.0	55.0 (53.3; 56.7)
Yemen (2013)	9,623	6.9	40.5 (38.7; 42.2)
**Eastern Europe and Central Asia**			
Albania (2017)	4,517	1.9	6.1 (5.2; 7.1)
Armenia (2015)	2,771	0.9	39.1 (37; 41.3)
Kyrgyzstan (2012)	3,078	3.0	61.0 (58.6; 63.3)
Tajikistan (2017)	3,934	2.9	50.4 (48.0; 52.7)
Turkey (2013)	5,369	1.9	59.6 (57.8; 61.4)
**South Asia**			
Afghanistan (2015)	13,144	6.3	39.4 (37.4; 41.5)
Bangladesh (2017)	13,986	10.0	70.3 (69.1; 71.4)
India (2019)	386,549	2.7	72.9 (72.7; 73.2)
Nepal (2016)	7,609	7.5	56.0 (54.3; 57.8)
Pakistan (2017)	5,996	5.5	48.2 (46.4; 50.1)
**East Asia and Pacific**			
Cambodia (2014)	7,970	4.1	56.0 (54.3; 57.8)
Indonesia (2017)	25,039	2.0	77.0 (76.2; 77.7)
Myanmar (2015)	5,228	3.0	74.7 (73.1; 76.3)
Papua New Guinea (2016)	6,806	4.5	47.7 (45.4; 50.0)
Philippines (2017)	10,998	3.1	55.3 (53.7; 56.9)
Timor Leste (2016)	3,849	3.1	45.4 (43.1; 47.7)
**Latin America and Caribbean**			
Colombia (2015)	21,535	10.3	85.6 (84.9; 86.3)
Dominican Republic (2013)	5,153	11.6	80.5 (78.7; 82.2)
Guatemala (2014)	11,716	8.7	65.4 (64.0; 66.6)
Haiti (2016)	6,516	7.9	41.8 (40.2; 43.5)
Honduras (2011)	11,765	11.0	75.9 (74.9; 76.9)
Peru (2020)	17,758	3.6	67.2 (65.9; 68.4)

Source: DHS 2010-2021.

mDFPS varied greatly across regions and groups of age. The lowest level of coverage was observed in West and Central Africa, where mDFPS was on average 28.9% among adolescents, and 35.7 and 32.8% among women aged 20–34 and 35–49 years, respectively. Latin America and the Caribbean was the region with the highest level of mDFPS, with average levels of coverage of 56.5, 69.2, and 72.3% among women aged 15–19, 20–34, and 35–49 years, respectively (Table 2). Public services were the main source of family planning in all regions, but they were less used by adolescents than adult women in most of the regions. The largest gap was identified in West and Central Africa, where 44.2% of the adolescents get their current method in public facilities while it was the source of family planning of 60.2% of the modern contraceptive users aged 20-−34 and 69.4% of those between the ages of 35 and 49. Similar patterns of method source among women of different groups of age were observed in the Middle East and North Africa, East Asia and the Pacific, and Latin America and the Caribbean (Table 2).

**Table 2:** Average demand for family planning satisfied by modern methods (mDFPS) and share of source of method according to women's age in low- and middle-income countries.

**Region**	**Women's age**	**mDFPS (%)**	**Source of method (%)**				
			**Public**	**Private**	**Non-profit**	**Friends/relatives**	**Other/unknown**
West and Central Africa	15–19	28.9	44.2	46.4	1.4	6.8	1.3
	20–34	35.7	60.2	33.8	2.0	3.0	1.0
	35–49	32.8	69.4	25.7	1.9	1.8	1.2
Eastern and Southern Africa	15–19	49.7	66.0	24.6	3.7	4.1	1.7
	20–34	61.2	73.4	20.7	3.9	0.8	1.1
	35–49	57.0	76.1	17.2	5.5	0.3	1.0
Middle East and North Africa	15–19	36.2	52.0	44.1	3.5	0.4	0.0
	20–34	56.8	51.2	42.2	6.3	0.1	0.2
	35–49	61.2	54.6	39.5	5.3	0.1	0.6
Eastern Europe and Central Asia	15–19	22.2	37.0	63.0	0.0	0.0	0.0
	20–34	41.2	52.7	46.7	0.1	0.2	0.4
	35–49	46.4	64.2	35.0	0.3	0.0	0.5
South Asia	15–19	34.5	36.5	56.0	1.5	5.8	0.1
	20–34	52.9	47.7	46.6	2.5	2.4	0.8
	35–49	64.9	61.4	33.7	2.3	0.9	1.7
East Asia and the Pacific	15–19	47.7	60.6	34.3	2.5	0.8	1.8
	20–34	61.3	60.6	36.6	1.4	0.3	1.1
	35–49	58.4	64.3	33.5	0.9	0.2	1.1
Latin America and the Caribbean	15–19	56.5	42.3	45.5	5.1	4.2	2.8
	20–34	69.2	45.7	39.6	9.6	1.3	3.8
	35–49	72.3	45.6	32.2	15.2	0.6	6.3

Source: DHS 2010-2021.

### 3.1. Demand for family planning satisfied and method source by women's age

**Figure 1:**
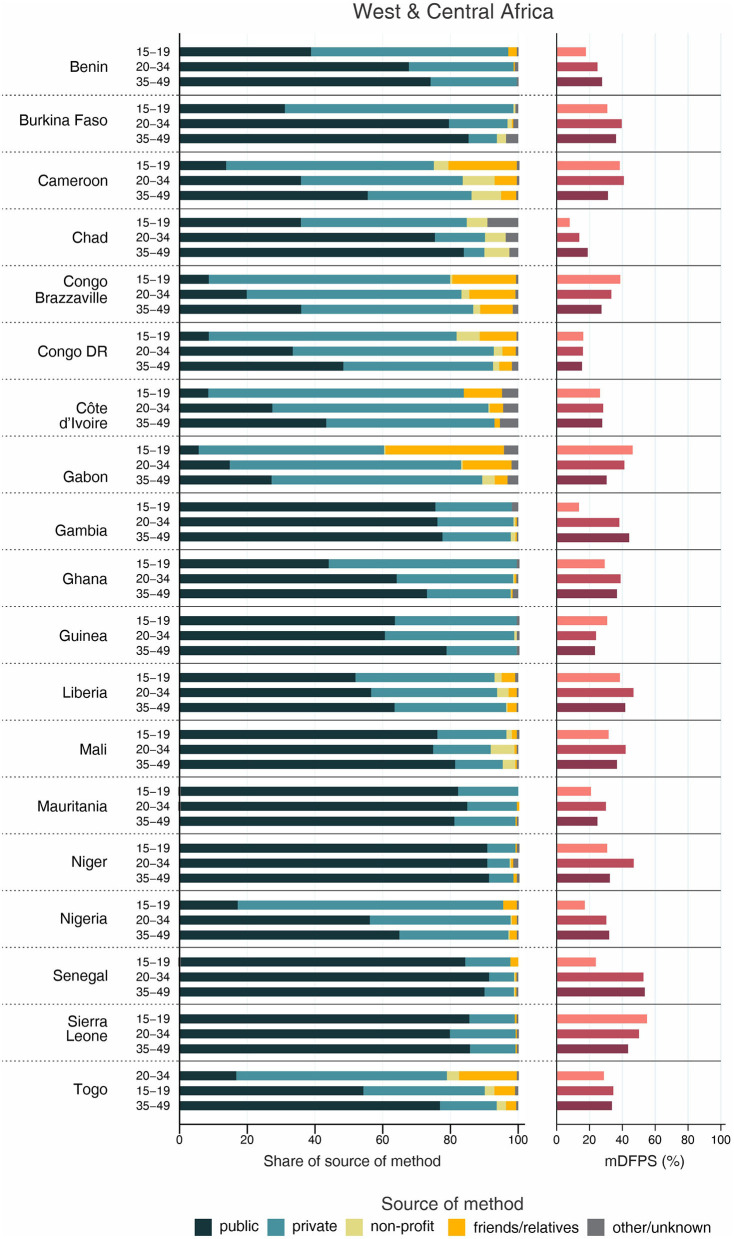
Demand for family planning satisfied by modern methods (mDFPS) and share of source of method by women's age in West and Central Africa. Source: DHS, 2010-2021.

**Figure 2:**
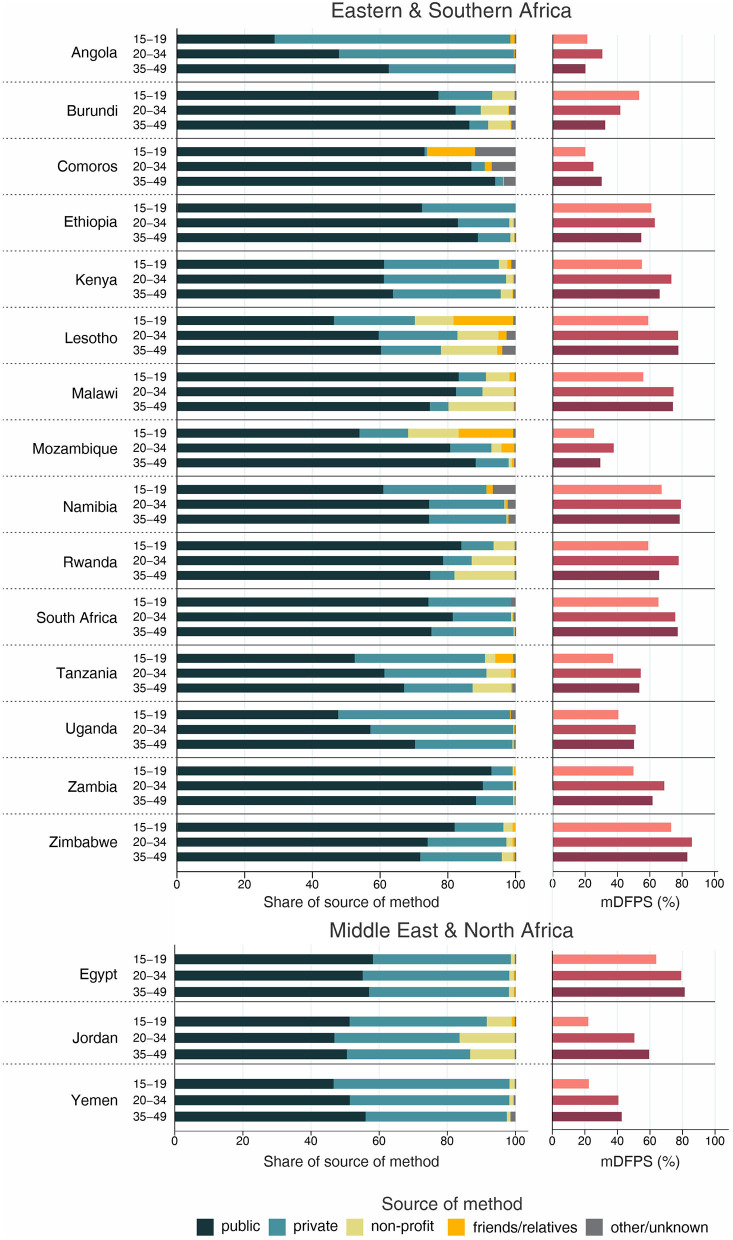
Demand for family planning satisfied by modern methods (mDFPS) and share of source of method by women's age in Eastern and Southern Africa and Middle East and North Africa. Source: DHS, 2010-2021.

**Figure 3:**
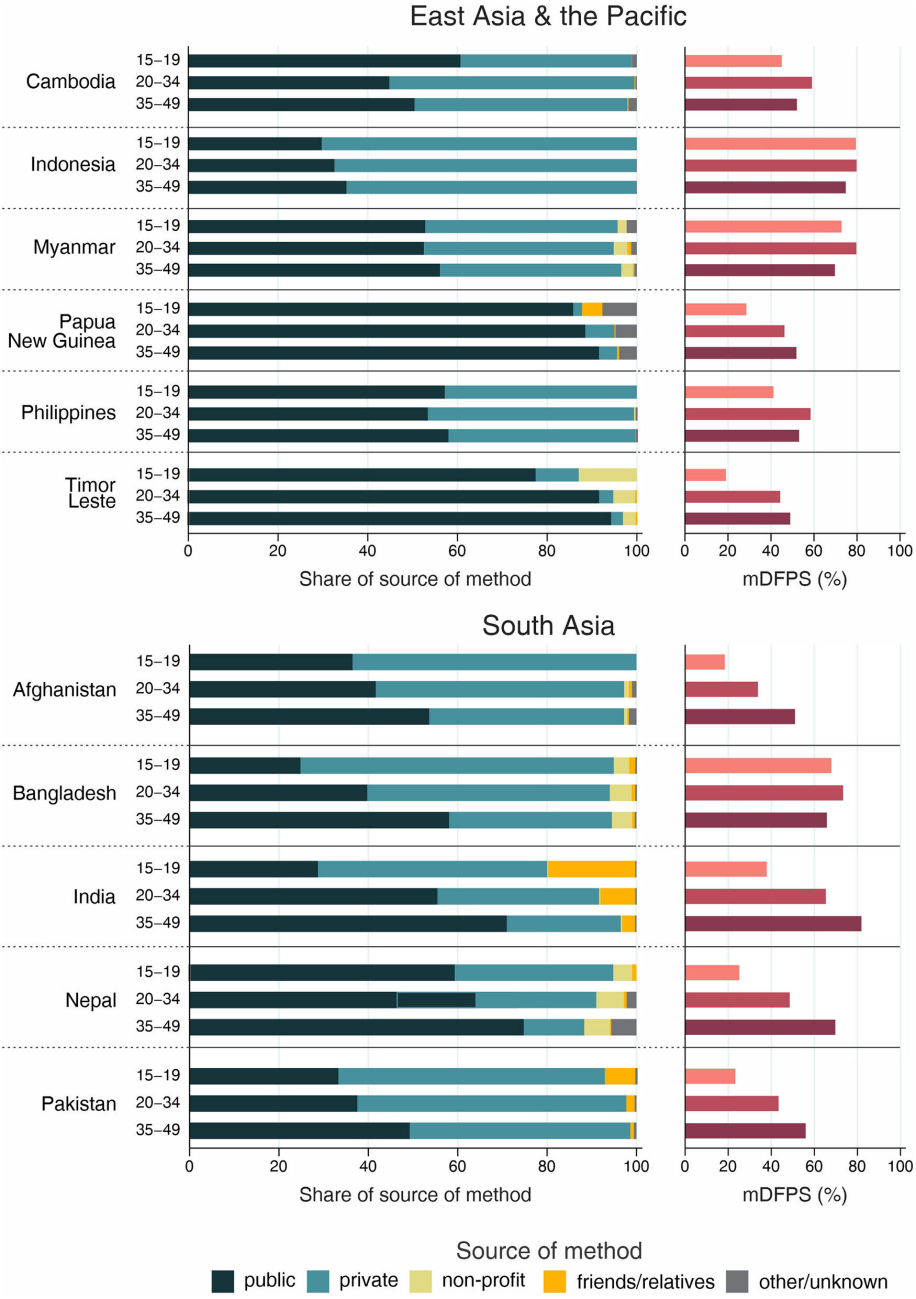
Demand for family planning satisfied by modern methods (mDFPS) and share of source of method by women's age in East Asia and the Pacific and South Asia. Source: DHS, 2010-2021.

**Figure 4:**
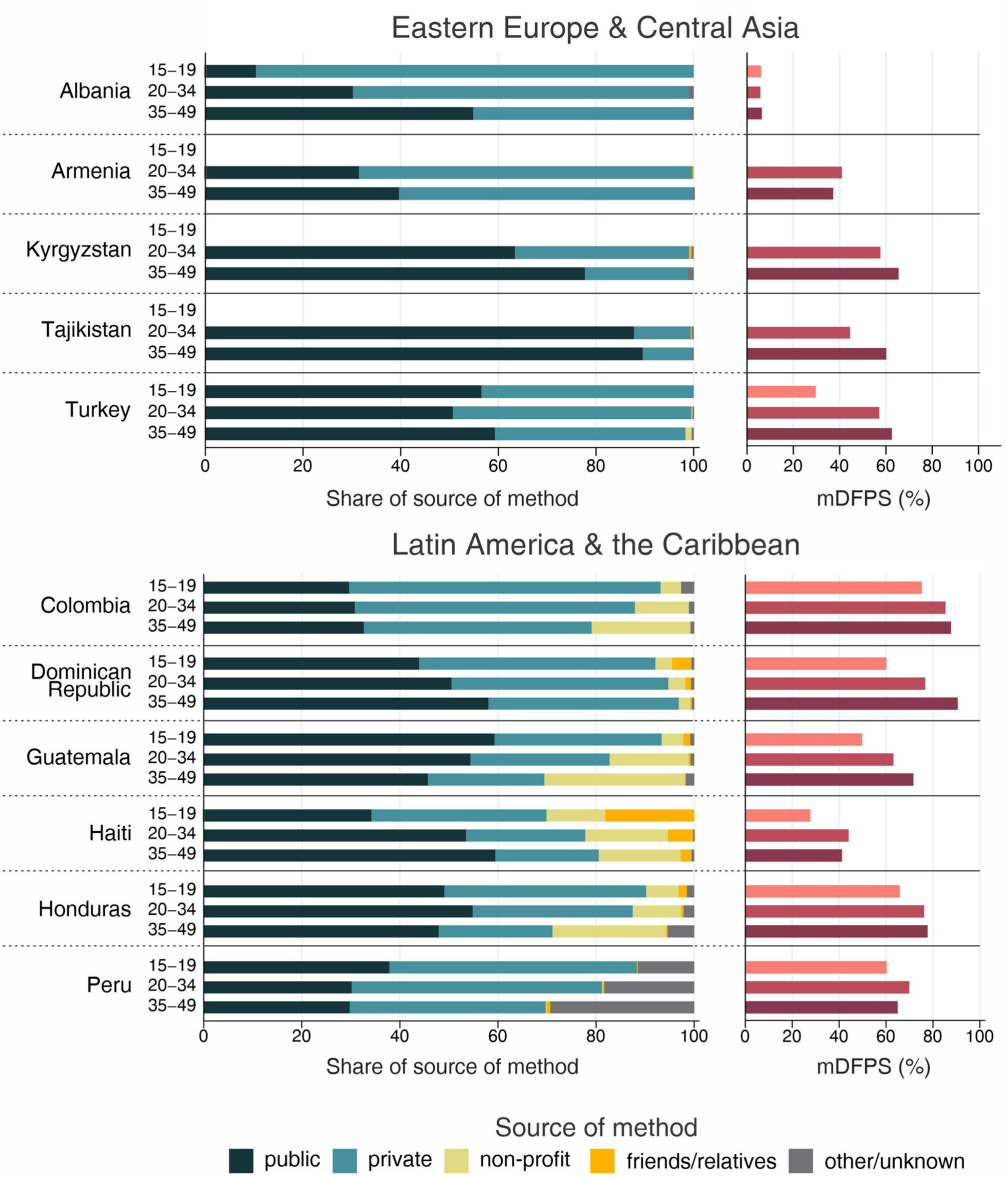
Demand for family planning satisfied by modern methods (mDFPS) and share of source of method by women's age in Eastern Europe and Central Asia and Latin America and the Caribbean. Source: DHS, 2010-2021. Bars of groups with fewer than 25 cases have been suppressed.

The country levels of mDFPS by age groups are presented in Figures 1–4. Our results indicated four patterns of inequality. The highest level of mDFPS was observed among young adults in 26 countries, among which larger gaps were usually observed in relation to adolescents with smaller gaps in comparison with older women. A monotonic increase with age was observed in 22 countries and a monotonic decrease with the increase of age in 5 countries. In Congo DR, Côte d'Ivoire, and Albania the difference by women's age was virtually null (Figures 1–4). For Armenia, Tajikistan, and Kyrgyzstan estimates on adolescents were suppressed due to insufficient sample size.

Adolescents presented much lower mDFPS than older women in a few countries from West and Central Africa, especially in Gambia and in Senegal, where mDFPS was 30 percentage points (p.p.) lower than among the older women. In Eastern and Southern Africa, the largest gaps were observed in Zambia, Malawi, Lesotho, and Tanzania. All three countries in the Middle East and North Africa presented large gaps according to women's age. Although we could not present estimates for adolescents for most of the countries from Eastern Europe and Central Asia, a very low level of coverage was observed among Albanian women from all age groups and a gap of more than 30 p.p. was identified between Turkish adolescents and women aged 35–49. In the other two Asian regions, adolescents presented lower mDFPS in 8 of the 11 countries, especially in India and Nepal, where their mDFPS was more than 40 p.p. lower than the coverage among the older women. Lower coverage among adolescents was identified in all countries in Latin America and the Caribbean, with larger gaps in the Dominican Republic (30 p.p.) and Haiti (16 p.p.) (Figures 1–4).

Regarding the source of method, our results indicated important differences across countries. A few countries stood out with a larger proportion of contraceptives obtained from friends or relatives. In all cases, adolescents relied more on this source than older women. Friends and relatives were a common source in six countries from West and Central Africa, where Gabon stood out with 35.0% of adolescents obtaining their contraceptives this way, followed by Cameroon (20.6%) and Congo Brazzaville (18.8%). It was also the case of three countries from Eastern and Southern Africa: Lesotho (17.6%), Mozambique (16.1%), and Comoros (14.2%). The adolescents' dependency on friends and relatives was also high in Haiti (18.1%) and India (19.4%) (Figures 1–4).

Significantly lower shares of the public sector among adolescents than among adult women were identified in 32 out of the 59 countries. The largest gaps were identified in Burkina Faso (where the public sector was the source of contraceptives of 31.1, 79.6, and 85.4%, of the contraceptive users aged 15–19, 20–34, and 35–49, respectively), in Togo (16.8, 54.3, and 76.9%), and Chad (35.9, 75.4, and 83.9%). In all of them, the private sector played a major role among adolescents. The public sector was highly used by women of all ages in some countries, such as Zambia, Senegal, Niger, and Mauritania, where it was the source of family planning for more than 80% of women. On the other hand, it was a less common source with the private sector playing a major role among women from all age groups in Côte d'Ivoire, Con go DR, Congo Brazzaville, and Gabon. In all these countries the public sector was the source of family planning for <10% of adolescents and <50% of adult women (Figures 1–4).

Non-profit services were relatively more used in Latin America and the Caribbean, especially among older women. The only exception was Peru, where the share of non-profit services was virtually null. In other regions, we did not observe a clear pattern of use of non-profit services by women's age (Figures 1–4).

### 3.2. Differences in the level of mDFPS and method source among married and unmarried adolescents

**Figure 5:**
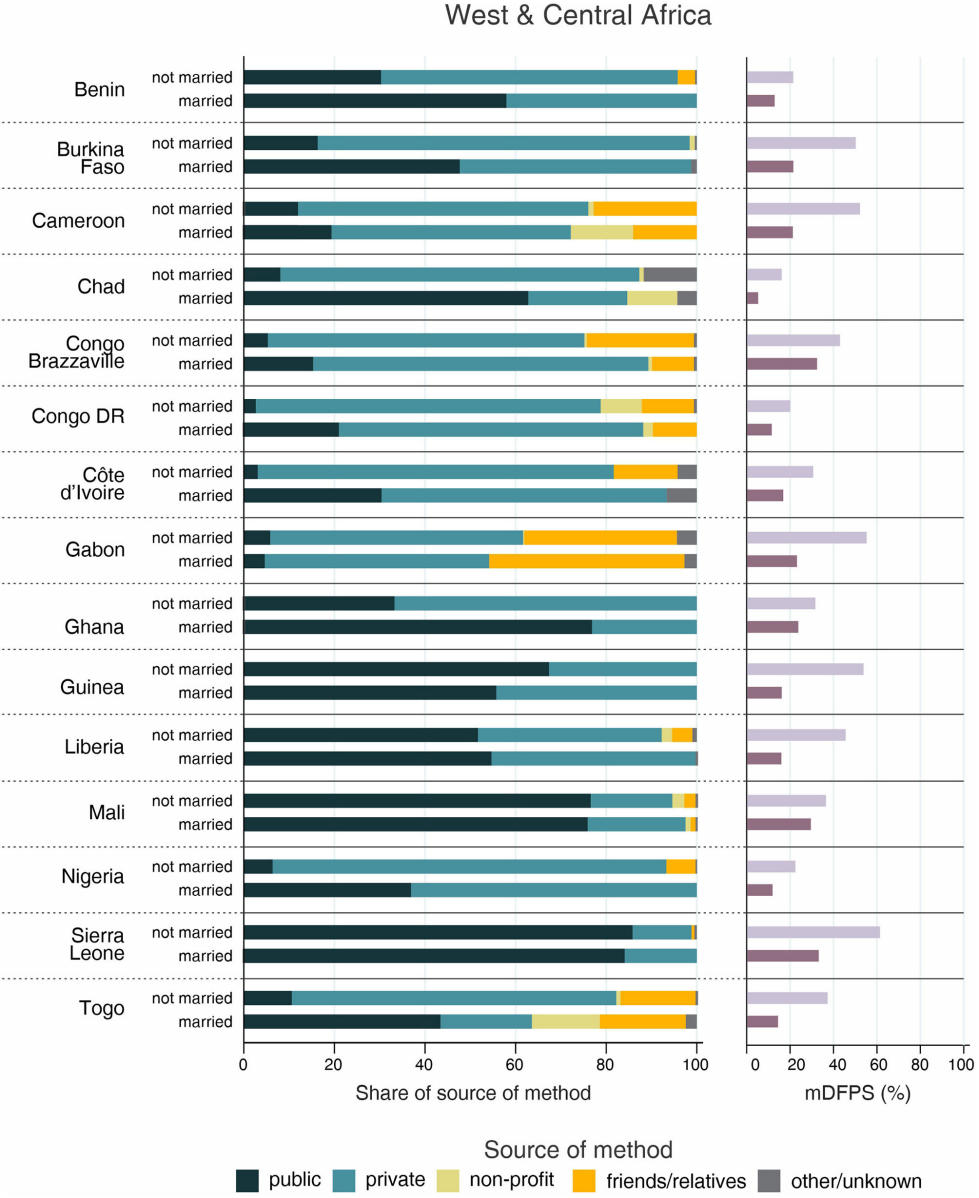
Demand for family planning satisfied by modern methods (mDFPS) and share of source of method by adolescents' marital status in West and Central Africa. Source: DHS, 2010-2021. Countries missing information on never married women or with fewer than 25 cases have been suppressed.

**Figure 6:**
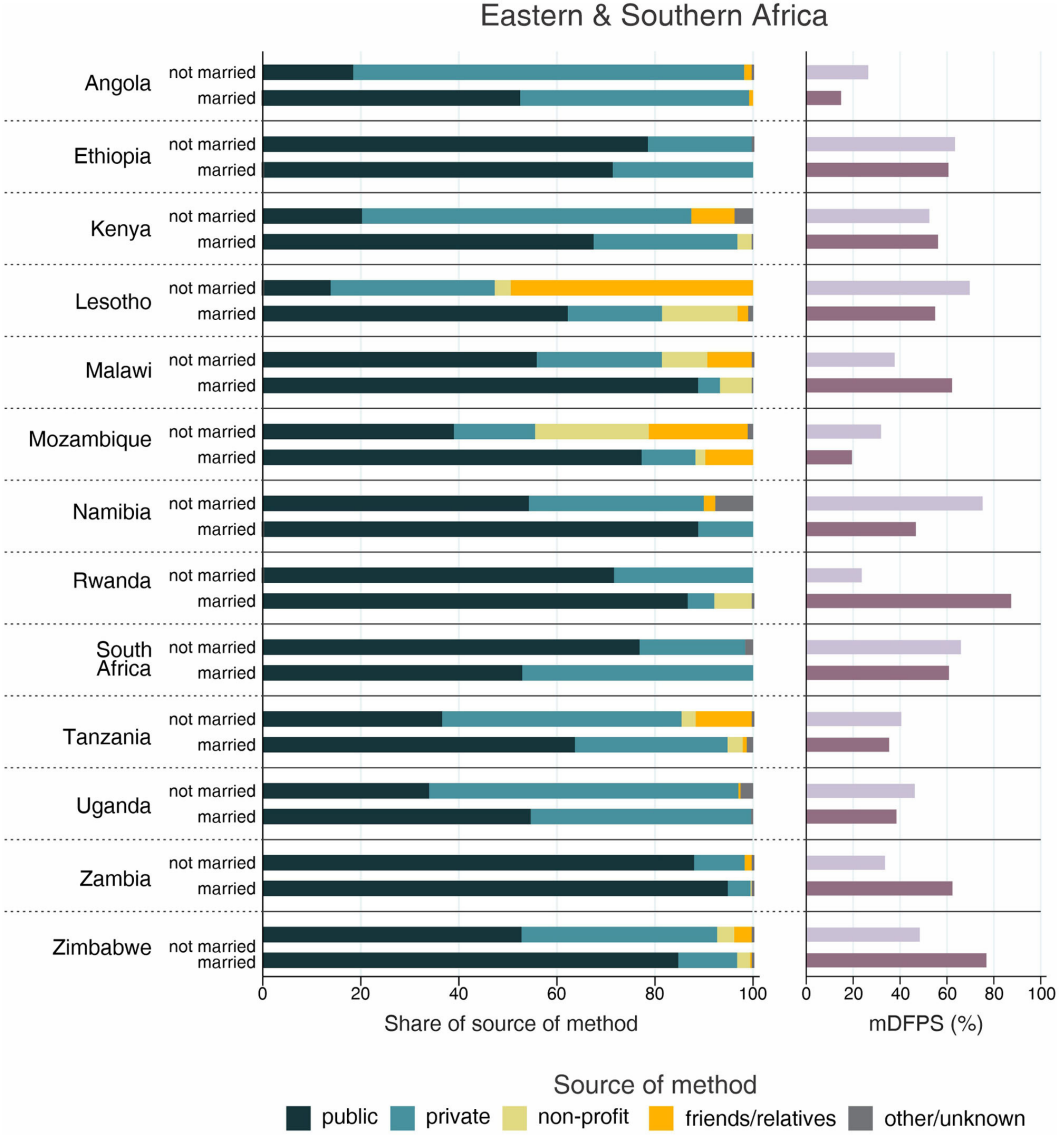
Demand for family planning satisfied by modern methods (mDFPS) and share of source of method by adolescent's marital status in Eastern and Southern Africa. Source: DHS, 2010-2021. Countries missing information on never married women or with fewer than 25 cases have been suppressed.

**Figure 7:**
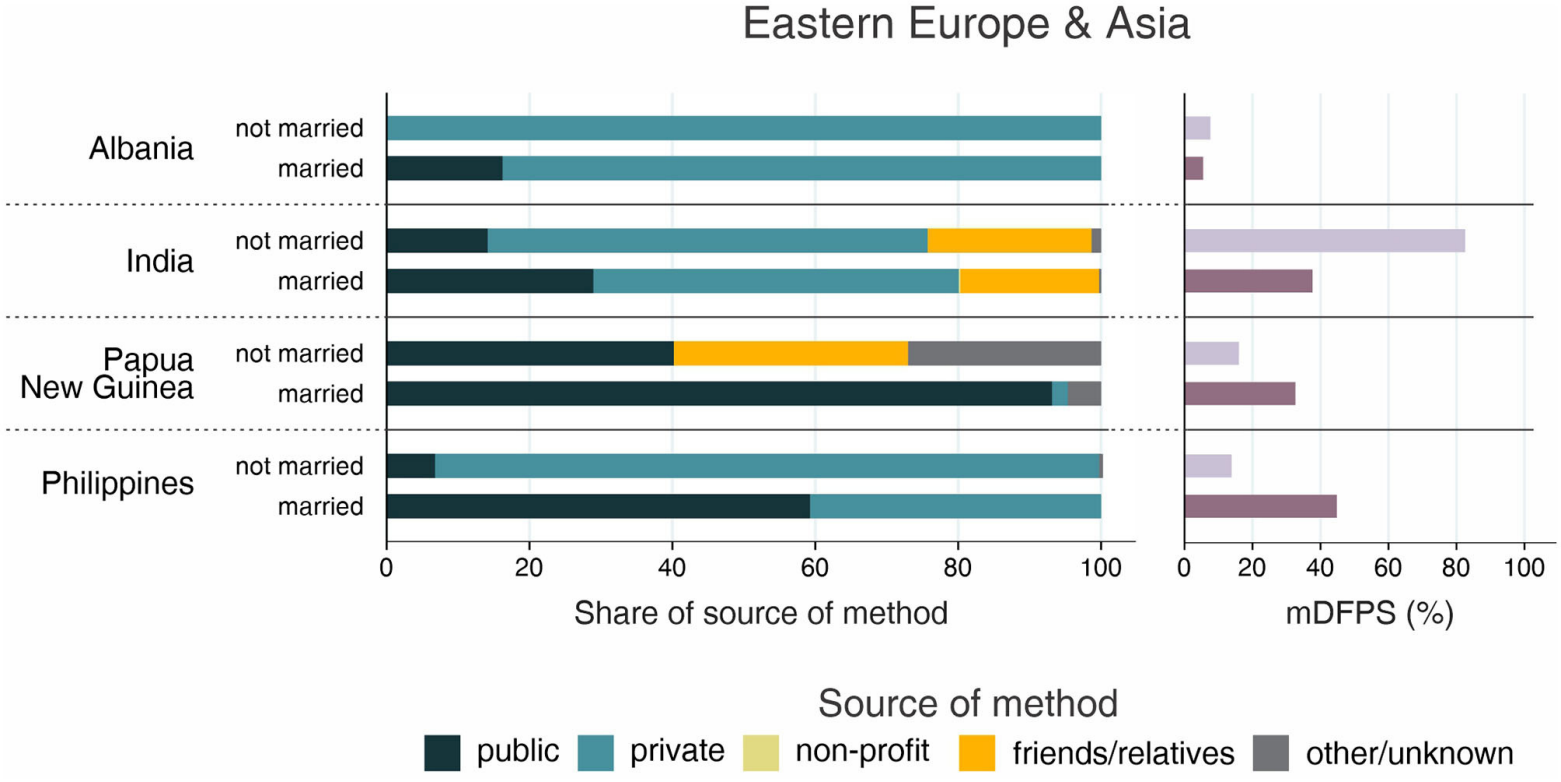
Demand for family planning satisfied by modern methods (mDFPS) and share of source of method by adolescent's marital status in Eastern Europe and Asia. Source: DHS, 2010-2021. Countries missing information on never married women or with fewer than 25 cases have been suppressed.

**Figure 8:**
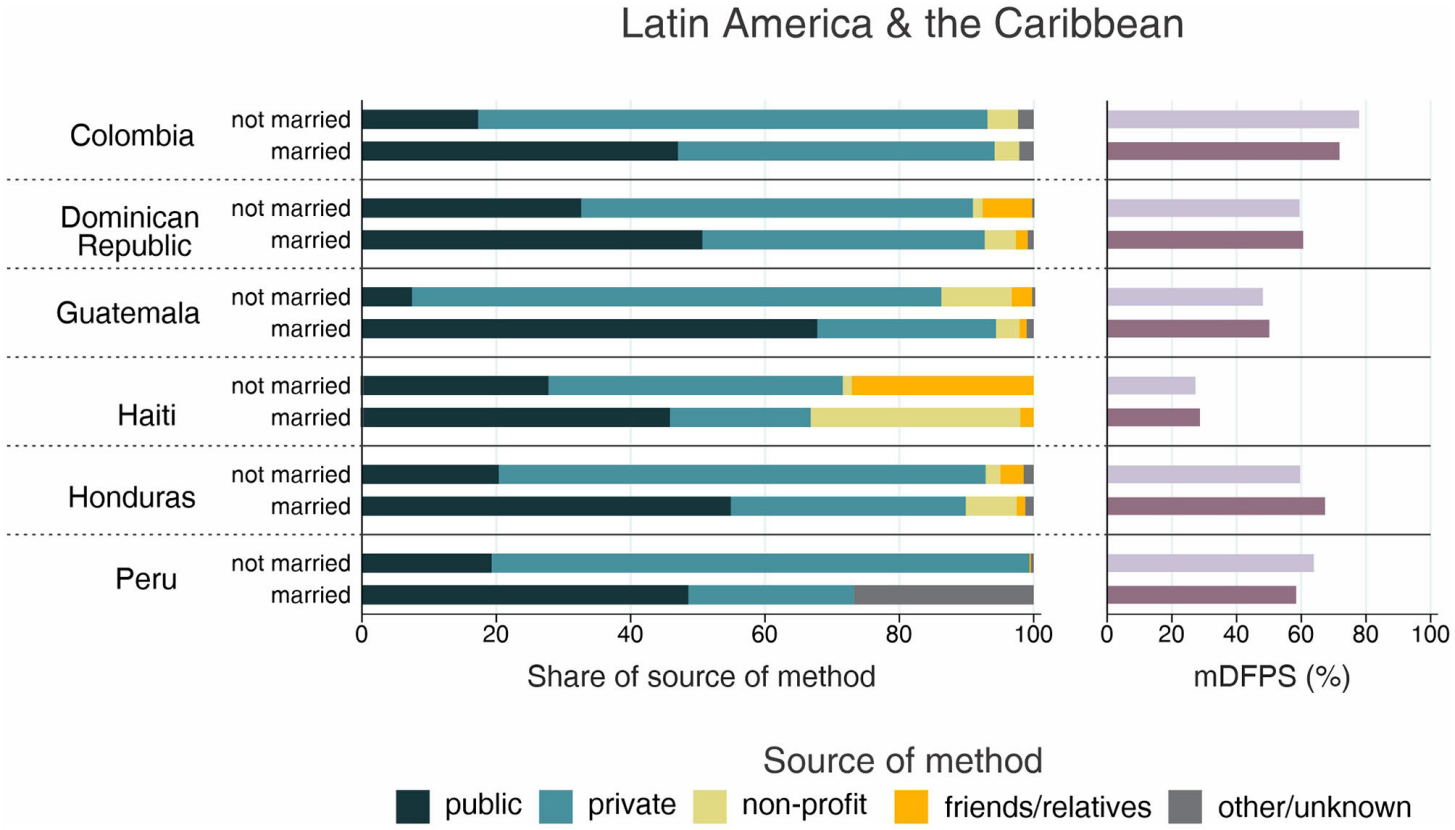
Demand for family planning satisfied by modern methods (mDFPS) and share of source of method by adolescent's marital status in Latin America and the Caribbean. Source: DHS, 2010-2021. Countries missing information on never married women or with fewer than 25 cases have been suppressed, or with fewer than 25 cases have been suppressed.

Inequalities in mDFPS and source of method by marital status of adolescents are shown in Figures 5–8. For 21 of the 59 countries, information on unmarried adolescents was either not available or the sample size was too small. This was especially true for Asian countries, where we were able to explore inequalities by marital status in only three out of 16 countries. Among the 38 countries with information, the patterns varied between and within regions. Unmarried adolescents presented significantly higher levels of mDFPS than married girls in 10 of the 38 countries with information. The largest differences between unmarried and married adolescents were found in Gabon (55.5 vs. 23.5%, respectively), Cameroon (52.5 vs. 21.5%), and Burkina Faso (50.5 vs. 21.8%). Married adolescents presented significantly higher mDFPS in five countries. The largest gaps were identified in Rwanda (23.7 vs. 87.4%) and Zambia (33.6 vs. 62.4%).

Regarding the share of method source, a clear pattern emerged about the use of friends or relatives as source of contraception. Although it was highly used by both married and unmarried adolescents in Gabon (43.1 vs. 33.7%, respectively), it was much more used among unmarried adolescents in almost all countries. The countries with the largest proportions of unmarried adolescents depending on friends or relatives were Lesotho (49.4%), Papua New Guinea (32.8%), and Haiti (27.1%). Large gaps were also identified in these three countries, being friends/relatives not used by married adolescents from Papua New Guinea and being it the source of contraceptives for only 2% of married adolescents from Lesotho and Haiti (Figures 5–8).

The share of the public sector was higher among married than among unmarried adolescents in 31 of 38 countries, among which we observed three different scenarios. In Gabon and Congo Brazzaville, our results indicate similar patterns of share of source regardless adolescents' marital status, with the public sector accounting for <20% of the mDFPS of both married and unmarried adolescents. In Ethiopia, Liberia, Mali, and Zambia the pattern of market share was also similar between married and unmarried adolescents, but with the public sector playing a major role as family planning provider. The last scenario was the more common, with higher use of the public sector only by married adolescents while unmarried adolescents relied mostly on the private sector and friends or relatives to get contraceptives (Figures 5–8).

Non-profit services were generally not used often and were a more important source in Haiti and Mozambique only. They were the source of contraceptives for 31.2% of unmarried adolescents in Haiti (compared to only 1.3% of married ones), while in Mozambique 23.2% of married adolescents relied on non-profit services compared to 2.0% of the unmarried ones (Figures 5–8).

### 3.3. Source of family planning not classified or not reported

The proportion of women who did not specify the source of the current contraceptive method used was negligible in most study countries (<2% overall in our sample). However, some countries presented a substantial proportion of “other” as the source. This was especially the case of Peru, where the proportion of “other” ranged between 11.5% among adolescents and 29.3% among women aged 35 to 49. In Comoros and Chad, the frequency of “other” among adolescents was 11.9 and 9.1%, respectively. When considering marital status, 27.0% of unmarried adolescents from Papua New Guinea, 26.7% of married adolescents from Peru, 14.8% of married adolescents from Comoros, and 11.7% of unmarried adolescents from Chad did not specify their last source of modern contraceptives.

## 4. Discussion

We used data from 59 low- and middle-income countries, analyzing a sample of 784,996 reproductive-age women to provide up-to-date estimates of mDFPS among women from different ranges of age, exploring differences in their source of family planning services and how level of mDFPS and source of method differ among married and unmarried adolescents. Our findings indicated that adolescents presented lower levels of mDFPS with relatively higher use of private facilities for family planning services compared to older women. We also found that mDFPS was higher among unmarried adolescents in most of the countries, but public facilities were much less used by them than by married adolescents. In almost all countries, unmarried adolescents relied in large part on private facilities and friends or relatives as source of contraceptives.

The lowest mDFPS among adolescents was found in West and Central Africa. Other studies have also documented lower levels of coverage in the region (3, 7, 12). Our findings indicated that, in addition to the large gap between adolescents and older women, mDFPS among married adolescents from West and Central Africa was half of the mDFPS among unmarried adolescents. This finding may be partly explained by the fact that the contraceptive method more used among unmarried adolescents is the male condom, which is almost not used by married girls (22). In addition, the region is marked by cultural norms of early age of sexual debut, early marriage, large spousal age gaps, and high adolescent birth rates (22, 23). Larger gaps between age groups were also found in the Asian region, especially in India and Nepal, countries where contraceptive use among adolescents is low and almost unchanged in the last years, where there is societal pressure to conceive soon after marriage, and where female permanent contraception is the method most used (24–26).

Looking at inequalities in mDFPS at the country level, we were able to identify the most extreme cases. Among the countries studied, the largest gap between married and unmarried adolescents was found in Rwanda, where mDFPS among married girls was almost 90% while it was lower than 25% among unmarried adolescents. This finding was surprising since the literature places Rwanda with high levels of family planning coverage, with a faster increase even among women from more vulnerable groups. Most of these results, however, consider only married women (3, 8, 27–29). Although the government has launched youth-friendly policies, premarital sex is uncommon in the country (30–33). It is a taboo covering up complex couples' dynamics and a decision-making process that can lead unmarried adolescents to risky sexual behavior (33). In a context of increasing rates of unwanted adolescent pregnancies and HIV infection, there is evidence that being unmarried is the more common reason for non-use of contraceptives among unmarried sexually active Rwandan adolescents (30, 32).

Regarding the inequalities in the source of method by women's age, our findings are consistent with previous studies that identified an overall lower share of the public sector among adolescents and young adults than among older women (19). However, we found similar shares of public and private sectors across groups of women's age in West and Central Africa, Middle East and North Africa, and in Latin America and the Caribbean. In these regions, the inequalities stood out when looking at adolescents' marital status, with a much lower share of the public sector among unmarried girls.

Looking at the country-level estimates, we were also able to identify important differences between countries. The countries included in our analysis vary greatly in terms of socioeconomic development, cultural norms, national willingness to invest in public health, health sector structure, and financing schemes. In almost all regions, the private sector was the main provider in some countries while it represented a minor proportion of the mDFPS in others. West and Central Africa presents huge heterogeneity between countries in terms of source of method. While there were countries with a higher share of the private sector among all women, such as Congo Brazzaville and Gabon, the private sector was a less representative source of family planning services in others, such as Mauritania, Niger, Senegal, and Sierra Leone. The same patterns were observed in terms of adolescents' marital status. Among countries with low use of the private sector for family planning services, it was previously documented in relation to other health needs (20), while among countries where the private services were largely used there is evidence of low satisfaction of users with government health services, with lack of technical competence being identified by the women (34). In addition, HIV/AIDS is still a major public health problem in several African countries. The higher share of the private sector may be also related to the higher share of male condoms, easily distributed in private pharmacies and markets (35, 36). Among the countries in Latin America and the Caribbean, although the differences in terms of share of public and private sectors by women's age were virtually null, large gaps were found looking at adolescents' marital status, with lower use of the public sector by unmarried adolescents in all countries. Haiti stood out as the country where friends or relatives were a more significant source, accounting for nearly 30% of the mDFPS among unmarried adolescents. This dependency on others to have access to family planning is unsurprising given there is evidence that half of the population has no access to healthcare and more than 70% of Haitian women still have limited availability of family planning services in both urban and rural areas (37, 38). The hundreds of NGOs working in the country do not seem to be able to offer enough services (39). In the Middle East and North Africa, the pattern of market share was also similar between and within countries, except for the higher use of non-profit services in Jordan. International organizations have been working in Jordan for several decades through partnerships with the government and direct provision of a full range of modern contraceptives in reproductive health clinics across the country (40–42). Although our findings are consistent with other studies that documented an overall low share of non-profit services (12), we found differences at regional and country levels. In addition to Jordan, this type of service was relatively more used in some Eastern and Southern African countries and in most of the countries in Latin America and the Caribbean, especially in Haiti.

Previous studies indicate that one of the methods more frequently used by adolescents is the male condom (43). Although the high share of friends or relatives as source of contraceptives may be a result of this higher role of the partner in the purchase of contraceptives, the inequality in the share of this source that we found in terms of marital status may partly result from lower accessibility of unmarried girls to family planning services. It is documented that boys find it easier to get condoms than girls (43), and that important reasons for the non-use of contraceptives by girls are related to provider attitudes, stigma, and shame (44, 45). In addition, although there is evidence that adolescents see their partners as people they could discuss family planning with, unreliable sources of family planning and sexual information, such as their peers and the internet, especially pornography websites, are highly declared by adolescents, since they considered it as more accessible (43, 45). These sources are, however, associated with misconceptions and incorrect information (43).

Among health institutions, despite the higher use of the private sector by adolescents, especially pharmacies and drug sellers which easily provide short-acting reversible methods, adolescents usually recognize public facilities as those of higher quality in terms of counseling and screening procedures (19, 46). Another study exploring factors related to the source of family planning services in 40 countries identified that differences in the source of method according to women's marital status vary according to the method chosen and to the marital status of the women (47). Similar source of contraceptives among married and unmarried women between the ages of 18 and 35 was identified among those using methods that require stronger training and are more frequently provided in hospitals and clinics, such as injectables and long-acting methods, and among the users of methods that can be easily obtained in shops in an accessible business transaction, such as male and female condoms. On the other hand, inequalities by marital status were observed concerning more expensive and self-administrated contraceptives, among which unmarried adolescents look for a discrete and no judgmental service while their married peers prefer public facilities that provide a free service and among whom the judgment barrier is nonexistent or much less expressive (47).

Although pharmacies and shops are a valuable source of short-acting contraceptives, they offer no provision of family planning counseling and knowledge on women's sexual and reproductive health, which are as important as the provision of contraceptives itself. Poor sexual and reproductive health education is associated with higher risks of sexual coercion, unintended pregnancies, induced abortions, and sexually transmitted infections (48, 49). In this sense, it is fundamental to consider the potential impact of the high use of these commercial sources by women at the beginning of their sexual and reproductive life.

Sexual and reproductive health strategies aiming to reach adolescents have been designed in the past years, resulting in an overall increase in demand for family planning satisfied among adolescents. However, unmarried adolescents are still under restrictive policies or cultural norms in several countries and public health services have not been provided to them or have not been properly provided (45). In addition, sex education is also scarce (45). To increase access and use of family planning services and reduce inequalities between married and unmarried adolescents, adolescent-friendly strategies must consider a provision of a wide range of free contraceptives or at reduced costs and the provision of reliable information on family planning knowledge, sexual and reproductive health, and girls' sexual empowerment. Means of family planning education that have been used successfully are communication through pamphlets and mobile phone technology and family planning integration with other health services used by adolescents (45).

Our study has some limitations. Information on source of method is available only in DHS surveys, therefore we have a low representation of some regions. In addition, we also have a low representation of countries where data on unmarried women was not collected or sexual activity among unmarried adolescents was rare or underreported. We were not able to evaluate inequalities by marital status in any of the countries from the Middle East and North Africa nor in most of those from Asia. Regarding the classification of the providers, although DHS has standardized the terminology in the more recent surveys, unclassified sources still occur. Women may be unsure how to classify unconventional health providers that may be public or non-profit. In addition, misclassification of the type of source still may occur when non-profit organizations work in partnership with the government or franchising private providers. Also, we were not able to identify potential impacts of public-private partnerships among which women may access family planning services in private facilities with reduced or no cost due to governmental subsidies. The other limitation in relation to the DHS methodology is that we were not able to access who was the friend/relative that provided the method. While some women who classified it as their source may receive their contraceptive from their boyfriend or husband, other women may depend on their peers or relatives. Another limitation is related to the lack of information on the quality of each type of provider since the only related information available in DHS is on side effects advice. Further research is needed to access the level of development of each sector in each country, the affordability of family planning services, and if the women choose that specific provider after suffering or to avoid suffering any kind of discrimination in the service she would prefer. We also have limitations related to the scope of our study. Since our main outcome is the share of method source, we opted to restrict demand for family planning satisfied to modern methods only. This restriction limits our interpretation on the role of traditional methods in satisfying the demand for family planning. There are also differences in the type of service that goes beyond our scope. Inequalities may exist in relation to the use of health services from different levels of quality, such as public hospitals and public health clinics. In addition, all the countries included have different ways on how the health system is organized, strategies to provide contraceptives, gender norms, and levels of economic development. The potential reasons for the differences we found were also not assessed.

## 5. Conclusion

Affordable access to high-quality health services is a fundamental human right. Our study brings light to the differences in mDFPS and share of method source by women's age and marital status in 59 low- and middle-income countries from all world regions. The inequalities identified suggest that the public sector of most of the countries included is still not reaching adolescents, especially adolescent girls who are not married. Our findings also highlight the importance of improving the services offered by the different health providers with specialized training of health workers, offering a full range of methods, and providing good and understandable information on women's health and family planning.

## Data availability statement

Publicly available datasets were analyzed in this study. This data can be found here: https://dhsprogram.com/.

## Author contributions

Conceptualization, data curation, methodology, visualization, and writing—review and editing: FH and AB. Formal analysis, investigation, and writing—original draft: FH. Funding acquisition and supervision: AB. Both authors have read and agreed to the published version of the manuscript.
