# Supplementary material for: What are the sources of contraceptives for married and unmarried adolescents: Health services or friends? Analysis of 59 low- and middle-income countries
Source: Front Public Health. 2023 Feb 6;11:1100129. doi: 10.3389/fpubh.2023.1100129 (PMC9939762; doi:10.3389/fpubh.2023.1100129)
Supplement: Supplementary file 1 [file Table_1.DOCX]

Supplementary Material

**Supplementary Table 1**. Demand for family planning satisfied by modern methods (mDFPS), unweighted sample size, and source of family planning among modern contraceptive users according to women’s age in 59 low- and middle-income countries.

| **Country** | **Age** | **mDFPS  % (95% CI)** | **Share of source of family planning  % (95%CI)** | | | | |
| --- | --- | --- | --- | --- | --- | --- | --- |
|  |  |  | Public | Private for-profit | Private non-profit | Friends/relatives | Other/unknown |
| **West & Central Africa** | | | | | | | |
| Benin (2017) | 15-19 | 18.1 (15.0; 21.7) | 38.9 (30.0; 48.6) | 58.2 (48.4; 67.4) | 0.0 | 2.6 (0.8; 8.2) | 0.3 (0.0; 1.7) |
|  | 20-34 | 25.2 (23.4; 27.1) | 67.7 (63.9; 71.4) | 31.1 (27.5; 34.9) | 0.0 | 0.2 (0.0; 0.8) | 1.0 (0.2; 1.8) |
|  | 35-49 | 27.9 (25.6; 30.4) | 74.2 (69.3; 78.4) | 25.6 (21.3; 30.3) | 0.0 | 0.0 | 0.3 (0.0; 0.8) |
| Burkina Faso (2010) | 15-19 | 31.1 (25.8; 37.0) | 31.1 (22.5; 41.2) | 67.5 (57.1; 76.4) | 0.6 (0.1; 4.0) | 0.0 | 0.8 (0.0; 3.5) |
|  | 20-34 | 39.9 (37.8; 42.1) | 79.6 (76.4; 82.4) | 17.3 (14.6; 20.4) | 1.3 (0.7; 2.6) | 0.4 (0.2; 0.9) | 1.4 (0.6; 2.2) |
|  | 35-49 | 36.4 (33.7; 39.3) | 85.4 (81.9; 88.3) | 8.4 (6.1; 11.3) | 2.7 (1.3; 5.4) | 0.0 | 3.6 (2.1; 5.1) |
| Cameroon (2018) | 15-19 | 38.7 (33.1; 44.6) | 13.8 (8.5; 21.6) | 61.3 (50.7; 71.0) | 4.3 (1.8; 9.8) | 20.6 (13.9; 29.5) | 0.0 |
|  | 20-34 | 41.2 (38.5; 43.9) | 35.9 (32.7; 39.3) | 47.7 (44.1; 51.4) | 9.4 (7.1; 12.4) | 6.9 (5.1; 9.1) | 0.1 (0.0; 0.3) |
|  | 35-49 | 31.5 (28.3; 34.9) | 55.6 (49.7; 61.4) | 30.7 (25.6; 36.2) | 8.7 (6.1; 12.3) | 4.5 (2.2; 8.9) | 0.6 (0.0; 1.5) |
| Chad (2014) | 15-19 | 8.2 (5.7; 11.7) | 35.9 (20.3; 55.1) | 49.0 (28.7; 69.6) | 6.1 (1.0; 29.8) | 0.0 | 9.1 (0.0; 19.2) |
|  | 20-34 | 14.1 (12.1; 16.3) | 75.4 (69.2; 80.7) | 14.8 (10.8; 19.9) | 6.1 (3.8; 9.7) | 0.0 | 3.7 (1.2; 6.2) |
|  | 35-49 | 19.2 (15.7; 23.3) | 83.9 (74.6; 90.3) | 6.1 (2.9; 12.2) | 7.4 (3.7; 14.3) | 0.0 | 2.6 (0.0; 5.3) |
| Congo Brazzaville (2011) | 15-19 | 39.0 (34.5; 43.8) | 8.6 (4.0; 17.8) | 71.3 (61.0; 79.8) | 0.6 (0.2; 2.4) | 18.8 (12.2; 27.9) | 0.6 (0.0; 2.7) |
|  | 20-34 | 33.6 (30.9; 36.4) | 19.9 (16.6; 23.5) | 63.4 (58.1; 68.4) | 2.3 (1.3; 3.9) | 13.6 (10.4; 17.7) | 0.8 (0.0; 2.1) |
|  | 35-49 | 27.6 (23.9; 31.7) | 35.9 (28.1; 44.6) | 50.8 (42.4; 59.2) | 2.1 (0.8; 5.1) | 9.6 (6.2; 14.6) | 1.6 (0.0; 4.0) |
| Congo Democratic Rep (2013) | 15-19 | 16.5 (12.7; 21.1) | 8.7 (4.1; 17.2) | 73.2 (58.5; 84.0) | 6.8 (1.2; 30.7) | 10.9 (5.5; 20.5) | 0.5 (0.0; 3.1) |
|  | 20-34 | 16.3 (14.4; 18.4) | 33.5 (26.7; 40.9) | 59.4 (51.9; 66.4) | 2.5 (1.2; 5.2) | 3.9 (2.5; 6.2) | 0.7 (0.0; 1.8) |
|  | 35-49 | 15.7 (13.2; 18.7) | 48.4 (38.9; 58.0) | 44.2 (35.4; 53.5) | 1.8 (0.7; 4.7) | 3.8 (1.8; 7.6) | 1.8 (0.0; 6.5) |
| Côte d'Ivoire (2011) | 15-19 | 26.7 (22.2; 31.7) | 8.5 (4.4; 15.9) | 75.4 (64.5; 83.8) | 0.0 | 11.3 (6.1; 20.0) | 4.8 (0.0; 13.6) |
|  | 20-34 | 28.6 (26.2; 31.2) | 27.4 (23.4; 31.8) | 63.9 (58.9; 68.6) | 0.3 (0.1; 1.1) | 3.9 (2.3; 6.7) | 4.4 (1.5; 7.3) |
|  | 35-49 | 28.0 (24.1; 32.3) | 43.3 (35.9; 51.1) | 49.7 (41.8; 57.5) | 0.0 | 1.6 (0.6; 4.4) | 5.4 (1.0; 9.8) |
| Gabon (2012) | 15-19 | 46.6 (38.3; 55.0) | 5.7 (2.8; 11.0) | 55.0 (45.3; 64.5) | 0.1 (0.0; 0.7) | 35.0 (25.8; 45.5) | 4.2 (0.0; 12.0) |
|  | 20-34 | 41.5 (38.4; 44.7) | 14.8 (11.0; 19.7) | 68.3 (62.3; 73.8) | 0.5 (0.1; 1.5) | 14.4 (10.2; 20.0) | 1.9 (1.2; 5.0) |
|  | 35-49 | 30.7 (26.2; 35.6) | 27.2 (18.8; 37.6) | 62.2 (52.7; 70.9) | 3.7 (1.3; 9.8) | 3.8 (2.0; 7.2) | 3.1 (0.0; 10.8) |
| Gambia (2019) | 15-19 | 13.9 (8.7; 21.4) | 75.6 (49.2; 90.8) | 22.6 (7.8; 50.1) | 0.0 | 0.0 | 1.8 (0.0; 6.5) |
|  | 20-34 | 38.5 (35.6; 41.4) | 76.2 (71.0; 80.7) | 22.5 (18.0; 27.6) | 0.6 (0.1; 2.5) | 0.5 (0.1; 1.7) | 0.3 (0.0; 0.8) |
|  | 35-49 | 44.4 (40.8; 48.1) | 77.6 (72.4; 82.1) | 20.2 (16.0; 25.2) | 1.4 (0.6; 3.2) | 0.4 (0.1; 1.7) | 0.4 (0.0; 1.2) |
| Ghana (2014) | 15-19 | 29.5 (21.9; 38.6) | 44.1 (30.1; 59.0) | 55.9 (41.0; 69.9) | 0.0 | 0.0 | 0.0 |
|  | 20-34 | 39.2 (36.3; 42.2) | 64.2 (59.2; 68.8) | 34.6 (30.2; 39.3) | 0.1 (0.0; 1.0) | 0.5 (0.2; 1.7) | 0.5 (0.0; 1.5) |
|  | 35-49 | 36.7 (32.8; 40.8) | 73.1 (68.5; 77.2) | 24.7 (20.7; 29.2) | 0.6 (0.2; 1.7) | 0.0 (0.0; 0.3) | 1.6 (0.0; 3.3) |
| Guinea (2018) | 15-19 | 31.0 (25.4; 37.3) | 63.6 (52.7; 73.3) | 36.4 (26.7; 47.3) | 0.0 | 0.0 | 0.0 |
|  | 20-34 | 24.3 (21.1; 27.7) | 60.7 (54.9; 66.1) | 38.2 (32.9; 43.8) | 1.1 (0.4; 2.9) | 0.0 | 0.0 |
|  | 35-49 | 23.6 (19.3; 28.6) | 78.9 (72.4; 84.2) | 20.9 (15.6; 27.5) | 0.2 (0.0; 1.3) | 0.0 | 0.0 |
| Liberia (2019) | 15-19 | 38.8 (33.4; 44.4) | 52.0 (41.2; 62.5) | 41.1 (31.6; 51.2) | 2.1 (0.5; 8.6) | 4.0 (1.2; 12.9) | 0.9 (0.0; 3.2) |
|  | 20-34 | 47.1 (42.7; 51.5) | 56.6 (51.8; 61.3) | 37.2 (32.2; 42.5) | 3.4 (1.8; 6.1) | 2.5 (1.3; 4.9) | 0.3 (0.0; 0.9) |
|  | 35-49 | 42.0 (38.2; 46.0) | 63.5 (56.4; 70.1) | 33.2 (27.0; 40.1) | 0.1 (0.0; 0.5) | 2.7 (1.1; 6.8) | 0.4 (0.0; 1.2) |
| Mali (2018) | 15-19 | 31.9 (26.7; 37.6) | 76.1 (67.1; 83.3) | 20.4 (13.6; 29.3) | 1.6 (0.5; 5.6) | 1.8 (0.4; 7.0) | 0.1 (0.0; 0.2) |
|  | 20-34 | 42.3 (39.2; 45.4) | 74.9 (70.3; 79.1) | 17.0 (14.0; 20.5) | 7.0 (4.6; 10.6) | 0.7 (0.3; 1.6) | 0.4 (0.0; 0.9) |
|  | 35-49 | 37.0 (33.3; 40.9) | 81.4 (77.0; 85.1) | 14.1 (10.8; 18.1) | 3.7 (2.2; 6.3) | 0.6 (0.2; 2.1) | 0.2 (0.0; 0.7) |
| Mauritania (2019) | 15-19 | 21.2 (16.4; 26.9) | 82.3 (69.8; 90.3) | 17.7 (9.7; 30.2) | 0.0 | 0.0 | 0.0 |
|  | 20-34 | 30.3 (27.9; 32.8) | 85.0 (81.8; 87.7) | 14.9 (12.2; 18.1) | 0.0 | 0.1 (0.0; 0.7) | 0.0 |
|  | 35-49 | 25.1 (22.3; 28.2) | 81.2 (75.5; 85.8) | 18.1 (13.5; 23.7) | 0.0 | 0.5 (0.1; 2.1) | 0.3 (0.0; 1.0) |
| Niger (2021) | 15-19 | 31.0 (22.6; 40.9) | 90.9 (71.9; 97.5) | 8.3 (2.0; 28.4) | 0.0 | 0.8 (0.1; 5.5) | 0 (0; 0) |
|  | 20-34 | 47.2 (42.6; 51.9) | 90.9 (83.4; 95.2) | 6.9 (3.2; 14.6) | 0.1 (0.0; 0.5) | 0.7 (0.2; 1.9) | 1.5 (0.2; 2.8) |
|  | 35-49 | 32.4 (27.5; 37.7) | 91.4 (86.2; 94.7) | 7.2 (4.3; 12.0) | 0.0 | 1.4 (0.3; 6.0) | 0.0 |
| Nigeria (2018) | 15-19 | 17.4 (14.1; 21.3) | 17.2 (10.6; 26.5) | 78.4 (68.4; 85.9) | 0.0 | 4.2 (1.6; 10.6) | 0.2 (0.0; 2.2) |
|  | 20-34 | 30.5 (28.7; 32.4) | 56.2 (52.9; 59.5) | 41.7 (38.6; 44.9) | 0.2 (0.1; 0.5) | 1.5 (0.9; 2.4) | 0.4 (0.0; 1.1) |
|  | 35-49 | 32.3 (30.7; 33.9) | 64.9 (60.4; 69.2) | 32.4 (28.2; 36.9) | 0.2 (0.1; 0.5) | 2.2 (1.4; 3.5) | 0.3 (0.0; 0.7) |
| Senegal (2019) | 15-19 | 24.2 (17.5; 32.4) | 84.4 (61.5; 94.8) | 13.3 (3.8; 37.5) | 0.0 | 2.3 (0.3; 15.1) | 0.0 |
|  | 20-34 | 53.1 (48.9; 57.3) | 91.4 (88.0; 93.9) | 7.6 (5.2; 11.0) | 0.1 (0.0; 0.9) | 0.4 (0.2; 0.8) | 0.5 (0.0; 1.3) |
|  | 35-49 | 53.9 (49.7; 58.1) | 90.1 (85.0; 93.6) | 9.0 (5.7; 13.8) | 0.1 (0.0; 0.7) | 0.3 (0.1; 1.1) | 0.5 (0.0; 1.4) |
| Sierra Leone (2019) | 15-19 | 55.3 (50.9; 59.7) | 85.6 (81.3; 89.0) | 13.4 (10.1; 17.6) | 0.0 | 0.5 (0.2; 1.4) | 0.4 (0.0; 1.1) |
|  | 20-34 | 50.4 (48.0; 52.8) | 79.9 (77.0; 82.5) | 19.4 (16.8; 22.3) | 0.0 | 0.5 (0.3; 1.0) | 0.2 (0.0; 0.5) |
|  | 35-49 | 43.8 (41.0; 46.6) | 85.8 (82.2; 88.7) | 13.4 (10.6; 16.9) | 0.0 | 0.5 (0.2; 1.4) | 0.3 (0.0; 0.7) |
| Togo (2013) | 15-19 | 29.1 (23.9; 34.8) | 16.8 (9.8; 27.2) | 62.2 (51.3; 72.0) | 3.6 (1.1; 11.3) | 17.2 (10.0; 28,0) | 0.2 (0.0; 1.3) |
|  | 20-34 | 34.8 (32.2; 37.4) | 54.3 (50.0; 58.5) | 35.9 (31.9; 40.1) | 2.8 (1.8; 4.4) | 6.1 (4.4; 8.4) | 0.9 (0; 1.8) |
|  | 35-49 | 33.9 (30.9; 37.1) | 76.9 (72.4; 80.8) | 16.8 (13.4; 20.8) | 2.7 (1.4; 5.2) | 3.0 (1.5; 6.2) | 0.6 (0.0; 1.6) |
| **Eastern & Southern Africa** | | | | | | | |
| Angola (2015) | 15-19 | 21.4 (17.3; 26.1) | 28.9 (20.4; 39.2) | 69.6 (59.4; 78.3) | 0.0 | 1.4 (0.4; 5.1) | 0.1 (0.0; 0.5) |
|  | 20-34 | 30.6 (27.3; 34.2) | 47.9 (42.3; 53.4) | 51.7 (46.1; 57.2) | 0.0 | 0.4 (0.1; 1.1) | 0.1 (0.0; 0.4) |
|  | 35-49 | 20.2 (16.5; 24.4) | 62.6 (53.8; 70.6) | 36.8 (28.7; 45.7) | 0.0 | 0.0 | 0.6 (0.0; 1.8) |
| Burundi (2016) | 15-19 | 53.4 (42.7; 63.9) | 77.2 (63.1; 87.1) | 15.9 (8.3; 28.3) | 6.9 (2.1; 20.2) | 0.0 | 0.0 |
|  | 20-34 | 41.8 (39.6; 43.9) | 82.3 (78.5; 85.6) | 7.5 (5.7; 9.8) | 8.3 (6.1; 11.1) | 0.1 (0.0; 0.7) | 1.9 (0.8; 3) |
|  | 35-49 | 32.4 (29.9; 35.0) | 86.4 (82.9; 89.2) | 5.5 (3.8; 8.0) | 6.7 (4.8; 9.4) | 0.2 (0.0; 1.2) | 1.2 (0.4; 2) |
| Comoros (2012) | 15-19 | 20.1 (13.0; 29.8) | 73.2 (52.1; 87.2) | 0.7 (0.1; 5.2) | 0.0 | 14.2 (4.1; 39.0) | 11.9 (0.5; 23.3) |
|  | 20-34 | 25.1 (22.1; 28.5) | 87.0 (81.5; 91.0) | 3.9 (1.9; 7.8) | 0.0 | 2.1 (0.7; 5.7) | 7.0 (3.7; 10.3) |
|  | 35-49 | 30.2 (25.0; 36.0) | 94.0 (88.3; 97.0) | 2.3 (0.7; 7.6) | 0.0 | 0.3 (0.0; 1.9) | 3.4 (0.4; 6.4) |
| Ethiopia (2016) | 15-19 | 61.0 (53.1; 68.4) | 72.4 (60.6; 81.7) | 27.6 (18.3; 39.4) | 0.0 | 0.0 | 0.0 |
|  | 20-34 | 63.1 (59.5; 66.6) | 83.0 (80.0; 85.6) | 15.2 (12.7; 18.0) | 1.4 (0.8; 2.3) | 0.0 (0.0; 0.1) | 0.4 (0.0; 0.8) |
|  | 35-49 | 54.7 (50.6; 58.8) | 88.9 (85.4; 91.6) | 9.6 (7.0; 13.0) | 1.1 (0.5; 2.4) | 0.3 (0.0; 2.0) | 0.1 (0.0; 0.2) |
| Kenya (2014) | 15-19 | 55.1 (47.1; 62.9) | 61.2 (52.8; 69.0) | 33.9 (26.5; 42.2) | 2.5 (0.6; 9.9) | 1.2 (0.3; 4.6) | 1.2 (0.0; 4.2) |
|  | 20-34 | 73.4 (71.5; 75.1) | 61.1 (59.2; 63.1) | 36.1 (34.1; 38.1) | 2.0 (1.4; 2.7) | 0.3 (0.2; 0.6) | 0.5 (0.2; 0.8) |
|  | 35-49 | 66.1 (63.6; 68.5) | 63.8 (61.4; 66.1) | 31.8 (29.6; 34.1) | 3.5 (2.7; 4.7) | 0.2 (0.1; 0.5) | 0.7 (0.3; 1.1) |
| Lesotho (2014) | 15-19 | 59.1 (51.8; 66.0) | 46.4 (35.4; 57.8) | 23.9 (16.8; 32.9) | 11.4 (6.4; 19.5) | 17.6 (10.9; 27.3) | 0.6 (0.0; 1.4) |
|  | 20-34 | 77.5 (75.2; 79.8) | 59.6 (56.0; 63.1) | 23.3 (20.5; 26.2) | 12.1 (9.9; 14.7) | 2.4 (1.6; 3.6) | 2.6 (1.6; 3.6) |
|  | 35-49 | 77.7 (74.3; 80.8) | 60.3 (55.5; 64.9) | 17.7 (14.6; 21.3) | 16.6 (13.3; 20.5) | 1.5 (0.8; 2.9) | 3.9 (2.3; 5.5) |
| Malawi (2015) | 15-19 | 56.0 (52.3; 59.6) | 83.2 (78.9; 86.8) | 8.0 (5.7; 11.3) | 7.0 (4.6; 10.3) | 1.6 (0.7; 3.5) | 0.2 (0.0; 0.6) |
|  | 20-34 | 74.7 (73.4; 76.0) | 82.5 (80.8; 84.0) | 7.8 (6.8; 9.0) | 9.3 (8.1; 10.7) | 0.2 (0.1; 0.3) | 0.2 (0.0; 0.4) |
|  | 35-49 | 74.3 (72.5; 76.0) | 74.7 (72.1; 77.2) | 5.5 (4.2; 7.2) | 19.4 (17.3; 21.7) | 0.0 (0.0; 0.3) | 0.4 (0.1; 0.7) |
| Mozambique (2011) | 15-19 | 25.6 (21.7; 29.9) | 53.9 (45.6; 62.1) | 14.4 (9.8; 20.7) | 14.9 (10.5; 20.8) | 16.1 (11.3; 22.4) | 0.7 (0.0; 1.8) |
|  | 20-34 | 37.7 (35.2; 40.3) | 80.7 (77.3; 83.7) | 12.2 (9.9; 14.9) | 3.0 (2.0; 4.4) | 3.8 (2.7; 5.3) | 0.3 (0.0; 0.6) |
|  | 35-49 | 29.4 (26.2; 32.8) | 88.3 (84.3; 91.3) | 9.7 (6.9; 13.4) | 1.0 (0.4; 2.3) | 0.7 (0.2; 2.1) | 0.4 (0.0; 1.0) |
| Namibia (2013) | 15-19 | 67.3 (60.2; 73.8) | 61.0 (52.3; 68.9) | 30.4 (22.9; 39.2) | 0.0 | 1.9 (0.7; 5.3) | 6.7 (1.5; 11.9) |
|  | 20-34 | 79.3 (76.9; 81.4) | 74.5 (71.3; 77.5) | 22.4 (19.5; 25.6) | 0.0 (0.0; 0.3) | 0.7 (0.4; 1.4) | 2.3 (0.9; 3.7) |
|  | 35-49 | 78.5 (75.9; 80.9) | 74.5 (70.3; 78.2) | 22.9 (19.1; 27.2) | 0.2 (0.0; 1.3) | 0.4 (0.1; 1.3) | 2.1 (0.9; 3.3) |
| Rwanda (2019) | 15-19 | 59.1 (47.3; 69.9) | 84.0 (68.6; 92.7) | 9.5 (3.8; 22.0) | 6.5 (1.6; 23.1) | 0.0 | 0.0 |
|  | 20-34 | 77.9 (76.1; 79.5) | 78.6 (76.7; 80.4) | 8.4 (7.1; 10.0) | 12.6 (11.2; 14.1) | 0.3 (0.1; 0.6) | 0.1 (0.0; 0.2) |
|  | 35-49 | 65.8 (63.7; 67.8) | 74.8 (72.4; 77.1) | 7.2 (5.9; 8.6) | 18.0 (16.0; 20.2) | 0.0 | 0.0 |
| South Africa (2016) | 15-19 | 65.4 (57.2; 72.7) | 74.3 (63.0; 83.1) | 24.4 (15.7; 35.8) | 0.0 | 0.0 | 1.4 (0.0; 3.9) |
|  | 20-34 | 75.7 (72.9; 78.4) | 81.4 (78.2; 84.3) | 17.2 (14.5; 20.3) | 0.7 (0.3; 1.6) | 0.1 (0.0; 0.4) | 0.6 (0.0; 1.3) |
|  | 35-49 | 77.3 (74.2; 80.1) | 75.2 (70.9; 79.0) | 24.3 (20.4; 28.6) | 0.4 (0.1; 2.1) | 0.0 | 0.1 (0.0; 0.3) |
| Tanzania (2015) | 15-19 | 37.3 (31.7; 43.3) | 52.5 (42.8; 62.0) | 38.5 (29.3; 48.7) | 3.0 (1.1; 8.3) | 5.3 (2.2; 12.3) | 0.7 (0.0; 2.8) |
|  | 20-34 | 54.4 (51.9; 56.8) | 61.3 (57.9; 64.6) | 30.2 (27.0; 33.5) | 7.2 (5.6; 9.2) | 1.1 (0.5; 2.1) | 0.3 (0.0; 0.7) |
|  | 35-49 | 53.5 (50.7; 56.3) | 67.1 (62.9; 71.0) | 20.3 (17.2; 23.7) | 11.3 (8.8; 14.4) | 0.4 (0.1; 1.3) | 0.9 (0.0; 2.1) |
| Uganda (2016) | 15-19 | 40.8 (36.4; 45.4) | 47.7 (40.2; 55.2) | 50.8 (43.1; 58.5) | 0.0 | 0.1 (0.0; 1.0) | 1.4 (0.0; 3.6) |
|  | 20-34 | 51.5 (49.5; 53.4) | 57.1 (54.1; 60.1) | 42.2 (39.3; 45.3) | 0.2 (0.1; 0.6) | 0.2 (0.1; 0.4) | 0.2 (0.0; 0.5) |
|  | 35-49 | 50.5 (48.1; 52.8) | 70.3 (67.3; 73.2) | 29.0 (26.1; 32.0) | 0.1 (0.0; 0.4) | 0.2 (0.1; 1.0) | 0.4 (0.0; 0.8) |
| Zambia (2018) | 15-19 | 49.9 (44.6; 55.3) | 92.9 (87.5; 96.1) | 6.3 (3.3; 11.6) | 0.3 (0.0; 2.4) | 0.5 (0.1; 3.4) | 0.0 |
|  | 20-34 | 69.0 (66.8; 71.1) | 90.4 (88.5; 92.0) | 8.8 (7.3; 10.6) | 0.6 (0.3; 1.4) | 0.1 (0.0; 0.3) | 0.1 (0.0; 0.2) |
|  | 35-49 | 61.7 (59.0; 64.3) | 88.3 (85.7; 90.5) | 11.0 (8.8; 13.6) | 0.4 (0.1; 1.3) | 0.0 | 0.3 (0.0; 0.6) |
| Zimbabwe (2015) | 15-19 | 73.2 (67.0; 78.6) | 82.0 (75.3; 87.2) | 14.4 (9.8; 20.7) | 2.7 (1.0; 7.4) | 0.9 (0.2; 3.5) | 0.0 |
|  | 20-34 | 86.1 (84.3; 87.6) | 74.0 (71.2; 76.7) | 23.3 (20.7; 26.0) | 1.8 (1.2; 2.8) | 0.7 (0.4; 1.1) | 0.2 (0.0; 0.5) |
|  | 35-49 | 83.2 (81.0; 85.2) | 71.9 (68.3; 75.3) | 24.1 (20.9; 27.6) | 3.3 (1.9; 5.6) | 0.7 (0.3; 1.4) | 0.1 (0.0; 0.2) |
| **Middle East & North Africa** | | | | | | | |
| Egypt (2014) | 15-19 | 63.9 (56.2; 70.9) | 58.2 (47.8; 67.9) | 40.5 (31.0; 50.8) | 1.3 (0.2; 8.9) | 0.0 | 0.0 |
|  | 20-34 | 79.3 (78.0; 80.5) | 55.2 (53.5; 56.9) | 43.0 (41.3; 44.8) | 1.3 (1.0; 1.7) | 0.3 (0.2; 0.6) | 0.2 (0.0; 0.4) |
|  | 35-49 | 81.4 (80.2; 82.5) | 57.1 (55.1; 59.0) | 41.0 (39.2; 42.9) | 1.5 (1.2; 2.0) | 0.2 (0.1; 0.4) | 0.2 (0.0; 0.4) |
| Jordan (2017) | 15-19 | 22.1 (13.0; 35.1) | 51.3 (25.2; 76.7) | 40.3 (16.3; 70.0) | 7.3 (2.0; 23.7) | 1.1 (0.1; 7.9) | 0.0 |
|  | 20-34 | 50.5 (47.9; 53.0) | 46.9 (43.4; 50.4) | 36.7 (33.5; 40.0) | 16.4 (13.8; 19.4) | 0.0 | 0.0 |
|  | 35-49 | 59.5 (57.3; 61.7) | 50.6 (47.3; 53.8) | 36.2 (33.1; 39.3) | 13.3 (11.1; 15.8) | 0.0 | 0.0 |
| Yemen (2013) | 15-19 | 22.5 (18.2; 27.4) | 46.7 (35.1; 58.6) | 51.6 (40.0; 63.1) | 1.7 (0.4; 6.9) | 0.0 | 0.0 |
|  | 20-34 | 40.6 (38.6; 42.7) | 51.4 (48.4; 54.5) | 46.8 (43.8; 49.9) | 1.2 (0.8; 2.0) | 0.0 | 0.5 (0.1; 0.9) |
|  | 35-49 | 42.6 (40.3; 45.0) | 56.0 (52.8; 59.3) | 41.5 (38.1; 44.9) | 1.1 (0.6; 2.0) | 0.0 | 1.4 (0.5; 2.3) |
| **Europe & Central Asia** | | | | | | | |
| Albania (2017) | 15-19 | 6.1 (2.6; 13.6) | 10.4 (1.3; 50.3) | 89.6 (49.7; 98.7) | 0.0 | 0.0 | 0.0 |
|  | 20-34 | 5.8 (4.6; 7.2) | 30.2 (20.8; 41.6) | 68.7 (57.0; 78.5) | 0.0 | 0.0 | 1.0 (0.0; 4.3) |
|  | 35-49 | 6.4 (5.2; 7.9) | 54.9 (44.9; 64.4) | 44.5 (34.9; 54.5) | 0.0 | 0.0 | 0.6 (0.0; 2.4) |
| Armenia (2015) | 15-19 | 33.7 (13.4; 62.7) | 0.0 | 100.0 | 0.0 | 0.0 | 0.0 |
|  | 20-34 | 41.0 (38.2; 43.9) | 31.5 (26.6; 36.8) | 68.3 (63.0; 73.2) | 0.0 | 0.2 (0.0; 1.7) | 0.0 |
|  | 35-49 | 37.2 (34.1; 40.4) | 39.6 (34.9; 44.6) | 60.2 (55.3; 65.0) | 0.0 | 0.1 (0.0; 0.8) | 0.0 |
| Kyrgyzstan (2012) | 15-19 | 33.2 (17.0; 54.7) | 51.9 (19.9; 82.4) | 48.1 (17.6; 80.1) | 0.0 | 0.0 | 0.0 |
|  | 20-34 | 57.6 (54.5; 60.8) | 63.4 (59.5; 67.2) | 35.7 (31.9; 39.7) | 0.0 | 0.5 (0.1; 1.9) | 0.4 (0.0; 1.0) |
|  | 35-49 | 65.5 (62.2; 68.6) | 77.7 (74.0; 81.0) | 21.2 (17.9; 24.8) | 0.0 | 0.0 | 1.1 (0.3; 1.9) |
| Tajikistan (2017) | 15-19 | 8.1 (1.9; 28.3) | 66.2 (10.8; 96.9) | 33.8 (3.1; 89.2) | 0.0 | 0.0 | 0.0 |
|  | 20-34 | 44.5 (41.9; 47.1) | 87.8 (85.1; 90.0) | 11.7 (9.5; 14.3) | 0.0 | 0.1 (0.0; 0.9) | 0.4 (0.0; 0.8) |
|  | 35-49 | 60.2 (56.7; 63.5) | 89.6 (87.1; 91.6) | 9.9 (7.9; 12.4) | 0.0 | 0.0 | 0.5 (0.0; 1.0) |
| Turkey (2013) | 15-19 | 29.7 (19.1; 43.1) | 56.5 (31.2; 78.9) | 43.5 (21.1; 68.9) | 0.0 | 0.0 | 0.0 |
|  | 20-34 | 57.1 (54.3; 59.9) | 50.7 (47.8; 53.6) | 48.9 (45.9; 51.8) | 0.3 (0.1; 0.8) | 0.0 | 0.2 (0.0; 0.6) |
|  | 35-49 | 62.6 (60.3; 64.8) | 59.3 (56.1; 62.4) | 39.0 (36.0; 42.1) | 1.3 (0.8; 2.1) | 0.0 | 0.4 (0.0; 0.9) |
| **South Asia** |  |  |  |  |  |  |  |
| Afghanistan (2015) | 15-19 | 18.4 (13.7; 24.4) | 36.5 (22.4; 53.4) | 63.5 (46.6; 77.6) | 0.0 | 0.0 | 0.0 |
|  | 20-34 | 33.8 (31.5; 36.2) | 41.7 (37.2; 46.3) | 55.6 (51.0; 60.1) | 1.0 (0.5; 2.2) | 0.7 (0.4; 1.5) | 1.0 (0.3; 1.7) |
|  | 35-49 | 51.0 (48.3; 53.8) | 53.7 (49.7; 57.6) | 43.6 (39.7; 47.5) | 0.8 (0.3; 1.7) | 0.3 (0.1; 0.8) | 1.7 (0.8; 2.6) |
| Bangladesh (2017) | 15-19 | 67.9 (64.6; 71.0) | 24.8 (21.7; 28.3) | 70.1 (66.6; 73.5) | 3.4 (2.3; 5.0) | 1.5 (0.8; 2.7) | 0.1 (0.0; 0.7) |
|  | 20-34 | 73.4 (72.0; 74.7) | 39.8 (38.0; 41.7) | 54.2 (52.4; 56.1) | 4.9 (4.1; 5.8) | 0.9 (0.7; 1.3) | 0.2 (0.0; 0.4) |
|  | 35-49 | 65.8 (64.2; 67.4) | 58.1 (55.8; 60.4) | 36.4 (34.3; 38.6) | 4.5 (3.7; 5.5) | 0.6 (0.3; 1.0) | 0.4 (0.1; 0.7) |
| India (2019) | 15-19 | 37.9 (36.2; 39.6) | 28.8 (25.8; 31.9) | 51.5 (48.3; 54.6) | 0.1 (0.1; 0.3) | 19.4 (17.3; 21.8) | 0.3 (0.1; 1.2) |
|  | 20-34 | 65.4 (64.9; 65.7) | 55.5 (55.0; 56.0) | 36.2 (35.7; 36.7) | 2.6 (2.2; 3.1) | 7.9 (7.6; 8.1) | 0.2 (0.1; 0.3) |
|  | 35-49 | 81.9 (81.6; 82.2) | 71.0 (70.6; 71.5) | 25.5 (25.1; 25.9) | 2.9 (2.5; 3.5) | 2.9 (2.8; 3.0) | 0.3 (0.2; 0.4) |
| Nepal (2016) | 15-19 | 25.1 (20.2; 30.8) | 59.3 (46.7; 70.8) | 35.5 (23.8; 49.3) | 4.2 (1.4; 12.4) | 0.9 (0.1; 6.2) | 0.0 |
|  | 20-34 | 48.5 (46.3; 50.8) | 64.0 (60.7; 67.1) | 27.1 (24.2; 30.2) | 6.1 (4.6; 8.2) | 0.6 (0.2; 1.9) | 2.2 (1.1; 3.3) |
|  | 35-49 | 69.7 (67.5; 71.9) | 74.8 (70.6; 78.6) | 13.5 (11.4; 16.0) | 5.9 (4.2; 8.4) | 0.1 (0.0; 0.4) | 5.6 (3.9; 7.3) |
| Pakistan (2017) | 15-19 | 23.3 (16.1; 32.6) | 33.3 (16.5; 55.8) | 59.6 (38.4; 77.7) | 0.0 | 7.0 (1.7; 25.3) | 0.0 |
|  | 20-34 | 43.4 (40.8; 46.0) | 37.6 (33.6; 41.7) | 60.2 (56.0; 64.2) | 0.0 | 1.9 (1.1; 3.3) | 0.4 (0.0; 1.1) |
|  | 35-49 | 56.0 (53.4; 58.5) | 49.3 (45.0; 53.6) | 49.4 (45.1; 53.7) | 0.0 | 0.8 (0.4; 1.3) | 0.6 (0.0; 1.4) |
| **East Asia & the Pacific** | | | | | | | |
| Cambodia (2014) | 15-19 | 45.0 (36.6; 53.7) | 60.7 (47.2; 72.8) | 38.2 (26.1; 51.9) | 0.0 | 0.0 | 1.1 (0.0; 4.0) |
|  | 20-34 | 59.1 (56.8; 61.3) | 44.9 (42.0; 47.8) | 54.6 (51.7; 57.4) | 0.0 | 0.2 (0.1; 0.6) | 0.3 (0.0; 0.7) |
|  | 35-49 | 52.1 (49.7; 54.4) | 50.5 (47.1; 53.9) | 47.4 (44.0; 50.9) | 0.0 | 0.2 (0.1; 0.7) | 1.9 (0.7; 3.1) |
| Indonesia (2017) | 15-19 | 79.5 (74.2; 83.9) | 29.8 (24.0; 36.3) | 70.2 (63.7; 76.0) | 0.0 | 0.0 | 0.0 |
|  | 20-34 | 79.8 (78.8; 80.8) | 32.6 (31.1; 34.2) | 67.3 (65.7; 68.8) | 0.0 | 0.0 | 0.1 (0.0; 0.3) |
|  | 35-49 | 74.8 (73.8; 75.7) | 35.3 (33.8; 36.8) | 64.6 (63.0; 66.1) | 0.0 | 0.0 | 0.1 (0.0; 0.2) |
| Myanmar (2015) | 15-19 | 72.8 (64.7; 79.6) | 52.9 (42.0; 63.5) | 42.9 (32.5; 54.0) | 2.0 (0.5; 7.6) | 0.0 | 2.3 (0.0; 5.6) |
|  | 20-34 | 79.7 (77.6; 81.7) | 52.6 (49.6; 55.5) | 42.3 (39.3; 45.3) | 3.0 (2.2; 4.2) | 0.9 (0.5; 1.5) | 1.3 (0.5; 2.1) |
|  | 35-49 | 69.7 (67.5; 71.9) | 56.1 (53.0; 59.2) | 40.4 (37.4; 43.6) | 2.5 (1.7; 3.7) | 0.3 (0.1; 0.8) | 0.6 (0.1; 1.1) |
| Papua New Guinea (2016) | 15-19 | 28.5 (20.3; 38.6) | 85.8 (70.0; 94.0) | 2.0 (0.3; 13.1) | 0.0 | 4.5 (1.1; 17.1) | 7.7 (0.0; 16.7) |
|  | 20-34 | 46.3 (43.6; 49.0) | 88.5 (85.9; 90.7) | 6.6 (4.9; 8.8) | 0.0 | 0.2 (0.1; 0.6) | 4.7 (3.4; 6) |
|  | 35-49 | 51.8 (48.4; 55.2) | 91.6 (89.4; 93.3) | 4.0 (2.9; 5.6) | 0.0 | 0.5 (0.1; 1.6) | 3.9 (2.7; 5.1) |
| Philippines (2017) | 15-19 | 41.1 (34.2; 48.4) | 57.2 (45.6; 68.0) | 42.8 (32.0; 54.4) | 0.0 | 0.0 | 0.0 |
|  | 20-34 | 58.4 (56.2; 60.6) | 53.4 (50.3; 56.5) | 46.0 (42.8; 49.1) | 0.3 (0.1; 1.0) | 0.2 (0.1; 0.6) | 0.1 (0.0; 0.3) |
|  | 35-49 | 53.1 (50.4; 55.7) | 58.0 (54.9; 61.0) | 41.8 (38.8; 44.9) | 0.1 (0.1; 0.3) | 0.0 (0.0; 0.2) | 0.0 |
| Timor Leste (2016) | 15-19 | 19.1 (11.1; 30.7) | 77.4 (42.6; 94.1) | 9.6 (1.4; 45.3) | 12.9 (1.9; 53.7) | 0.0 | 0.0 |
|  | 20-34 | 44.3 (41.2; 47.4) | 91.6 (88.5; 93.9) | 3.2 (2.0; 4.9) | 4.8 (3.2; 7.3) | 0.4 (0.1; 1.1) | 0.0 |
|  | 35-49 | 48.9 (45.8; 52.1) | 94.3 (90.4; 96.6) | 2.6 (1.1; 6.4) | 3.0 (1.5; 5.7) | 0.1 (0.0; 0.9) | 0.0 |
| **Latin America & the Caribbean** | | | | | | | |
| Colombia (2015) | 15-19 | 75.3 (72.6; 77.8) | 29.7 (26.5; 33.1) | 63.5 (59.6; 67.2) | 4.2 (3.0; 5.8) | 0.0 | 2.7 (0.6; 4.8) |
|  | 20-34 | 85.4 (84.3; 86.4) | 30.8 (29.2; 32.4) | 57.2 (55.5; 58.8) | 11.0 (9.9; 12.2) | 0.0 | 1.0 (0.6; 1.4) |
|  | 35-49 | 87.7 (86.5; 88.9) | 32.7 (30.6; 34.8) | 46.5 (44.1; 48.9) | 20.2 (18.5; 21.9) | 0.0 | 0.7 (0.3; 1.1) |
| Dominican Republic (2013) | 15-19 | 60.2 (54.3; 65.7) | 43.9 (36.8; 51.3) | 48.2 (40.7; 55.8) | 3.4 (1.5; 7.7) | 3.9 (1.7; 8.7) | 0.5 (0.0; 1.5) |
|  | 20-34 | 76.7 (74.2; 79.0) | 50.6 (46.7; 54.4) | 44.2 (40.6; 47.9) | 3.5 (2.5; 4.7) | 1.2 (0.7; 1.9) | 0.6 (0.0; 1.3) |
|  | 35-49 | 90.6 (88.4; 92.4) | 58.1 (54.1; 61.9) | 38.8 (35.1; 42.7) | 2.3 (1.4; 3.7) | 0.3 (0.1; 1.4) | 0.4 (0.0; 1.0) |
| Guatemala (2014) | 15-19 | 49.8 (45.6; 54.1) | 59.3 (53.3; 65.1) | 34.0 (28.6; 40.0) | 4.5 (2.7; 7.2) | 1.4 (0.5; 3.7) | 0.8 (0.0; 2.3) |
|  | 20-34 | 63.2 (61.6; 64.7) | 54.4 (52.5; 56.3) | 28.4 (26.7; 30.1) | 16.1 (14.6; 17.8) | 0.4 (0.2; 0.8) | 0.7 (0.4; 1.0) |
|  | 35-49 | 71.7 (69.8; 73.5) | 45.7 (43.5; 48.0) | 23.7 (21.8; 25.8) | 28.7 (26.6; 30.9) | 0.1 (0.0; 0.3) | 1.7 (1.1; 2.3) |
| Haiti (2016) | 15-19 | 27.8 (23.6; 32.3) | 34.2 (25.8; 43.7) | 35.7 (27.0; 45.4) | 12.0 (7.4; 18.7) | 18.1 (12.1; 26.2) | 0.0 |
|  | 20-34 | 44.1 (41.9; 46.2) | 53.5 (49.7; 57.3) | 24.3 (21.3; 27.5) | 16.9 (14.5; 19.6) | 5.2 (3.9; 7.0) | 0.1 (0.0; 0.3) |
|  | 35-49 | 41.2 (38.7; 43.8) | 59.5 (54.9; 63.8) | 21.1 (17.3; 25.4) | 16.7 (13.8; 20.1) | 2.2 (1.1; 4.2) | 0.5 (0.0; 0.1) |
| Honduras (2011) | 15-19 | 65.9 (62.3; 69.3) | 49.1 (44.6; 53.5) | 41.2 (36.9; 45.6) | 6.6 (4.6; 9.5) | 1.7 (0.9; 3.0) | 1.5 (0.0; 3.1) |
|  | 20-34 | 76.2 (74.9; 77.5) | 54.8 (53.0; 56.7) | 32.6 (30.9; 34.4) | 9.9 (8.7; 11.2) | 0.5 (0.3; 0.8) | 2.1 (1.4; 2.8) |
|  | 35-49 | 77.7 (76.1; 79.3) | 47.9 (45.9; 50.0) | 23.2 (21.4; 25.1) | 23.2 (21.4; 25.1) | 0.2 (0.1; 0.6) | 5.4 (4.3; 6.5) |
| Peru (2020) | 15-19 | 60.3 (53.3; 66.9) | 37.9 (31.3; 44.9) | 50.6 (41.4; 59.7) | 0.0 | 0.1 (0.0; 0.5) | 11.5 (5.9; 17.1) |
|  | 20-34 | 69.9 (68.2; 71.6) | 30.2 (28.1; 32.4) | 51.2 (48.3; 54.1) | 0.0 | 0.3 (0.1; 0.5) | 18.3 (15.4; 21.2) |
|  | 35-49 | 65.0 (63.1; 66.8) | 29.8 (27.4; 32.3) | 40.1 (36.8; 43.6) | 0.0 | 0.8 (0.3; 2.0) | 29.3 (25.9; 32.7) |

**Supplementary Table 2**. Demand for family planning satisfied by modern methods (mDFPS), unweighted sample size, and source of family planning among modern contraceptive users according to women’s marital status in 59 low- and middle-income countries.

| **Country** | **Marital status** | **mDFPS  % (95% CI)** | **Share of source of family planning  % (95%CI)** | | | | |
| --- | --- | --- | --- | --- | --- | --- | --- |
|  |  |  | Public | Private for-profit | Private non-profit | Friends/relatives | Other/unknown |
| **West & Central Africa** | | | | | | | |
| Benin (2017) | Married | 13.2 (9.5; 18.0) | 58.0 (39.1; 74.8) | 42.0 (25.2; 60.9) | 0.0 | 0.0 | 0.0 |
|  | Not married | 21.7 (17.3; 26.8) | 30.3 (21.0; 41.6) | 65.5 (54.0; 75.5) | 0.0 | 3.8 (1.2; 11.7) | 0.4 (0.3; 0.4) |
| Burkina Faso (2010) | Married | 21.8 (16.2; 28.7) | 47.7 (32.2; 63.6) | 51.1 (34.8; 67.1) | 0.0 | 0.0 | 1.2 (1.2; 1.2) |
|  | Not married | 50.5 (40.4; 60.6) | 16.3 (8.5; 29.1) | 82.2 (69.3; 90.4) | 1.1 (0.2; 7.4) | 0.0 | 0.4 (0.4; 0.4) |
| Cameroon (2018) | Married | 21.5 (16.1; 28.1) | 19.3 (9.6; 35.1) | 52.9 (36.2; 68.9) | 13.7 (4.9; 32.8) | 14.0 (4.8; 34.7) | 0.0 |
|  | Not married | 52.5 (45.6; 59.3) | 12.0 (6.5; 21.1) | 64.1 (52.2; 74.5) | 1.2 (0.3; 5.0) | 22.8 (14.9; 33.2) | 0.0 |
| Chad (2014) | Married | 5.6 (3.3; 9.1) | 62.8 (35.7; 83.7) | 21.9 (6.8; 51.9) | 11.1 (1.6; 48.8) | 0.0 | 4.3 (4.2; 4.3) |
|  | Not married | 16.4 (9.5; 26.7) | 8.1 (1.1; 41.1) | 79.2 (45.4; 94.6) | 1.0 (0.1; 7.1) | 0.0 | 11.7 (11.5; 11.7) |
| Congo Brazzaville (2011) | Married | 32.7 (26.4; 39.6) | 15.3 (5.6; 35.2) | 74.1 (56.6; 86.2) | 0.8 (0.2; 3.9) | 9.2 (4.0; 20.0) | 0.6 (0.6; 0.6) |
|  | Not married | 43.3 (36.9; 50.0) | 5.3 (1.5; 16.7) | 69.9 (57.1; 80.2) | 0.5 (0.1; 3.9) | 23.6 (14.7; 35.8) | 0.6 (0.6; 0.6) |
| Congo Dem Rep (2013) | Married | 11.8 (8.1; 17.1) | 21.0 (9.2; 41.0) | 67.2 (45.7; 83.3) | 2.1 (0.3; 14.2) | 9.7 (2.1; 34.8) | 0.0 |
|  | Not married | 20.4 (14.5; 27.9) | 2.6 (0.8; 8.7) | 76.2 (54.6; 89.4) | 9.1 (1.3; 42.5) | 11.5 (4.9; 24.5) | 0.6 (0.6; 0.6) |
| Côte d'Ivoire (2011) | Married | 17.1 (11.0; 25.6) | 30.4 (13.7; 54.5) | 63.0 (36.9; 83.2) | 0.0 | 0.0 | 6.6 (6.4; 6.6) |
|  | Not married | 30.9 (25.2; 37.2) | 3.1 (1.0; 9.2) | 78.6 (66.3; 87.2) | 0.0 | 14.1 (7.6; 24.7) | 4.2 (4.1; 4.2) |
| Gabon (2012) | Married | 23.5 (15.9; 33.3) | 4.6 (1.8; 11.5) | 49.6 (29.9; 69.4) | 0.0 | 43.1 (24.6; 63.8) | 2.7 (2.6; 2.7) |
|  | Not married | 55.5 (45.0; 65.6) | 5.8 (2.7; 12.2) | 56.0 (45.4; 66.1) | 0.1 (0; 0.9) | 33.7 (24.0; 45.1) | 4.4 (4.3; 4.4) |
| Gambia (2019) | Married | 13.0 (7.8; 20.8) | 83.7 (62.0; 94.2) | 14.0 (4.5; 36.4) | 0.0 | 0.0 | 2.3 (2.2; 2.3) |
|  | Not married | 33.3 (11.9; 64.8) | 8.7 (0.6; 60.6) | 91.3 (39.4; 99.4) | 0.0 | 0.0 | 0.0 |
| Ghana (2014) | Married | 24.1 (14.4; 37.5) | 76.9 (53.1; 90.7) | 23.1 (9.3; 46.9) | 0.0 | 0.0 | 0.0 |
|  | Not married | 31.9 (22.9; 42.5) | 33.3 (18.3; 52.6) | 66.7 (47.4; 81.7) | 0.0 | 0.0 | 0.0 |
| Guinea (2018) | Married | 16.4 (10.1; 25.4) | 55.8 (30.8; 78.1) | 44.2 (21.9; 69.2) | 0.0 | 0.0 | 0.0 |
|  | Not married | 54.2 (44.7; 63.4) | 67.4 (57.2; 76.1) | 32.6 (23.9; 42.8) | 0.0 | 0.0 | 0.0 |
| Liberia (2019) | Married | 16.3 (9.8; 25.7) | 54.7 (30.5; 76.8) | 45.3 (23.2; 69.5) | 0.0 | 0.0 | 0.0 |
|  | Not married | 45.9 (39.0; 52.9) | 51.7 (40.1; 63.1) | 40.6 (30.6; 51.4) | 2.3 (0.5; 9.5) | 4.5 (1.3; 14.2) | 1.0 (0.9; 1.0) |
| Mali (2018) | Married | 29.8 (23.6; 36.9) | 75.9 (64.7; 84.4) | 21.6 (13.5; 32.8) | 1.1 (0.3; 4.3) | 1.3 (0.2; 8.7) | 0.1 (0.1; 0.1) |
|  | Not married | 36.8 (26.6; 48.3) | 76.6 (58.3; 88.5) | 18.0 (7.8; 36.3) | 2.6 (0.4; 16.9) | 2.8 (0.4; 17.7) | 0 |
| Mauritania (2019) | Married | 21.6 (16.7; 27.4) | 82.3 (69.8; 90.3) | 17.7 (9.7; 30.2) | 0.0 | 0.0 | 0.0 |
|  | Not married | 0.0 | 0.0 | 0.0 | 0.0 | 0.0 | 0.0 |
| Niger (2021) | Married | 29.8 (21.8; 39.1) | 97.2 (82.3; 99.6) | 2.8 (0.4; 17.7) | 0.0 | 0.0 | 0.0 |
|  | Not married | 81.5 (30.6; 97.8) | 0.0 | 88.0 (31.0; 99.2) | 0.0 | 12 (0.8; 69) | 0.0 |
| Nigeria (2018) | Married | 12.2 (8.6; 17.1) | 36.9 (21.5; 55.6) | 63.1 (44.4; 78.5) | 0.0 | 0.0 | 0.0 |
|  | Not married | 22.7 (17.3; 29.2) | 6.4 (2.7; 14.3) | 86.9 (76.5; 93.2) | 0.0 | 6.5 (2.5; 16.1) | 0.2 (0.2; 0.2) |
| Senegal (2019) | Married | 24.6 (17.8; 33.0) | 84.1 (61.0; 94.7) | 13.5 (3.8; 38.0) | 0.0 | 2.4 (0.3; 15.3) | 0.0 |
|  | Not married | 11.2 (0.9; 64.5) | 100 | 0.0 | 0.0 | 0.0 | 0.0 |
| Sierra Leone (2019) | Married | 33.5 (26.0; 41.8) | 84.1 (72.5; 91.3) | 15.9 (8.7; 27.5) | 0.0 | 0.0 | 0.0 |
|  | Not married | 61.7 (56.7; 66.5) | 85.9 (81.3; 89.4) | 13.0 (9.6; 17.5) | 0.0 | 0.6 (0.2; 1.7) | 0.5 (0.5; 0.5) |
| Togo (2013) | Married | 14.7 (8.9; 23.3) | 43.4 (22.7; 66.7) | 20.2 (7.9; 43.0) | 15.0 (3.6; 45.6) | 19 (4.9; 51.8) | 2.4 (2.3; 2.4) |
|  | Not married | 37.6 (30.4; 45.4) | 10.6 (4.7; 22.1) | 71.7 (59.6; 81.3) | 0.9 (0.1; 6.1) | 16.8 (9.3; 28.6) | 0 |
| **Eastern & Southern Africa** | | | | | | | |
| Angola (2015) | Married | 14.9 (9.9; 21.7) | 52.5 (32.6; 71.6) | 46.7 (27.8; 66.7) | 0.0 | 0.8 (0.1; 5.6) | 0.0 |
|  | Not married | 26.4 (21.0; 32.7) | 18.5 (10.6; 30.3) | 79.7 (67.9; 88.0) | 0.0 | 1.7 (0.4; 7.3) | 0.1 (0.1; 0.1) |
| Burundi (2016) | Married | 54.6 (42.8; 66.0) | 83.8 (68.4; 92.6) | 8.3 (3.1; 20.5) | 7.8 (2.3; 23.7) | 0.0 | 0.0 |
|  | Not married | 48.6 (27.0; 70.7) | 47.3 (20.3; 75.9) | 50.2 (22.1; 78.2) | 2.5 (0.3; 16.4) | 0.0 | 0.0 |
| Comoros (2012) | Married | 18.0 (11.4; 27.2) | 76.2 (57.1; 88.5) | 0.9 (0.1; 6.7) | 0.0 | 8.0 (1.7; 31.1) | 14.8 (14.7; 14.8) |
|  | Not married | 39.2 (12.5; 74.5) | 60.9 (9.6; 95.8) | 0.0 | 0.0 | 39.1 (4.2; 90.4) | 0.0 |
| Ethiopia (2016) | Married | 60.7 (52.0; 68.6) | 71.4 (58.9; 81.4) | 28.6 (18.6; 41.1) | 0.0 | 0.0 | 0.0 |
|  | Not married | 63.5 (41.6; 80.9) | 78.6 (47.1; 93.8) | 21.4 (6.2; 52.9) | 0.0 | 0.0 | 0.0 |
| Kenya (2014) | Married | 56.2 (46.5; 65.5) | 67.5 (58.5; 75.3) | 29.3 (21.9; 38.1) | 2.9 (0.7; 11.3) | 0.0 | 0.3 (0.3; 0.3) |
|  | Not married | 52.5 (37.4; 67.2) | 20.3 (9.6; 37.8) | 67.2 (45.9; 83.1) | 0.0 | 8.8 (2.2; 29.7) | 3.8 (3.6; 3.8) |
| Lesotho (2014) | Married | 55.0 (46.2; 63.5) | 62.2 (49.0; 73.9) | 19.2 (11.5; 30.4) | 15.4 (8.4; 26.5) | 2.1 (0.5; 8.2) | 1.0 (1.0; 1.0) |
|  | Not married | 69.7 (56.5; 80.3) | 13.9 (6.1; 28.4) | 33.4 (21.5; 47.9) | 3.3 (0.5; 20.1) | 49.4 (33.0; 66.0) | 0.0 |
| Malawi (2015) | Married | 62.2 (57.9; 66.3) | 88.8 (84.5; 92.1) | 4.5 (2.6; 7.6) | 6.5 (4.1; 10.1) | 0.0 | 0.2 (0.2; 0.2) |
|  | Not married | 37.7 (30.5; 45.6) | 55.9 (43.4; 67.7) | 25.5 (16.3; 37.6) | 9.3 (4.3; 18.8) | 9.3 (4.3; 19.2) | 0.0 |
| Mozambique (2011) | Married | 19.5 (14.8; 25.4) | 77.3 (64.9; 86.2) | 11.0 (4.9; 22.9) | 2 (0.4; 9.0) | 9.7 (5.2; 17.6) | 0.0 |
|  | Not married | 31.9 (25.6; 39.0) | 39.0 (29.4; 49.6) | 16.6 (10.6; 24.9) | 23.2 (16.6; 31.4) | 20.2 (13.4; 29.2) | 1.1 (1.1; 1.1) |
| Namibia (2013) | Married | 46.7 (33.8; 60.1) | 88.9 (68.8; 96.6) | 11.1 (3.4; 31.2) | 0.0 | 0.0 | 0.0 |
|  | Not married | 75.2 (68.1; 81.2) | 54.3 (44.7; 63.6) | 35.7 (26.7; 45.8) | 0.0 | 2.4 (0.8; 6.5) | 7.7 (7.6; 7.7) |
| Rwanda (2019) | Married | 87.4 (70.6; 95.3) | 86.7 (68.2; 95.2) | 5.5 (1.3; 20.5) | 7.9 (1.9; 27.3) | 0.0 | 0.0 |
|  | Not married | 23.7 (12.8; 39.7) | 71.7 (38.1; 91.2) | 28.3 (8.8; 61.9) | 0.0 | 0.0 | 0.0 |
| South Africa (2016) | Married | 60.9 (33.0; 83.1) | 52.9 (23.5; 80.5) | 47.1 (19.5; 76.5) | 0.0 | 0.0 | 0.0 |
|  | Not married | 66.0 (57.5; 73.6) | 76.9 (65.2; 85.5) | 21.6 (13.1; 33.4) | 0.0 | 0.0 | 1.6 (1.5; 1.6) |
| Tanzania (2015) | Married | 35.3 (28.8; 42.5) | 63.7 (50.5; 75.1) | 31.1 (20.1; 44.9) | 3.1 (1.0; 9.2) | 0.8 (0.1; 5.8) | 1.3 (1.2; 1.3) |
|  | Not married | 40.6 (31.5; 50.3) | 36.6 (22.9; 52.9) | 48.8 (34.2; 63.6) | 2.9 (0.4; 18.2) | 11.7 (4.5; 27.0) | 0.0 |
| Uganda (2016) | Married | 38.5 (33.4; 43.8) | 54.7 (45.5; 63.6) | 44.9 (36.0; 54.2) | 0.0 | 0.0 | 0.4 (0.4; 0.4) |
|  | Not married | 46.3 (37.8; 55.0) | 34 (24.2; 45.3) | 63.1 (50.9; 73.8) | 0.0 | 0.4 (0.1; 3.0) | 2.5 (2.5; 2.5) |
| Zambia (2018) | Married | 62.4 (54.7; 69.4) | 94.9 (89; 97.7) | 4.6 (2.0; 10.6) | 0.5 (0.1; 3.4) | 0.0 | 0.0 |
|  | Not married | 33.6 (26.8; 41.1) | 88 (73.8; 95) | 10.3 (3.9; 24.7) | 0.0 | 1.7 (0.2; 11.0) | 0.0 |
| Zimbabwe (2015) | Married | 76.8 (70.3; 82.3) | 84.7 (77.6; 89.9) | 12.0 (7.5; 18.7) | 2.6 (0.9; 7.9) | 0.6 (0.1; 4.0) | 0.0 |
|  | Not married | 48.4 (32.2; 64.9) | 52.8 (31.8; 72.9) | 39.9 (20.9; 62.5) | 3.5 (0.5; 21.4) | 3.8 (0.5; 23.0) | 0.0 |
| **Middle East & North Africa** | | | | | | | |
| Egypt (2014) | Married | 63.9 (56.2; 70.9) | 58.2 (47.8; 67.9) | 40.5 (31.0; 50.8) | 1.3 (0.2; 8.9) | 1.1 (0.1; 7.9) | 0.0 |
|  | Not married | NA | NA | NA | NA | NA | NA |
| Jordan (2017) | Married | 22.1 (13.0; 35.1) | 51.3 (25.2; 76.7) | 40.3 (16.3; 70.0) | 7.3 (2.0; 23.7) | 0.0 | 0.0 |
|  | Not married | NA | NA | NA | NA | NA | NA |
| Yemen (2013) | Married | 22.5 (18.2; 27.4) | 46.7 (35.1; 58.6) | 51.6 (40.0; 63.1) | 1.7 (0.4; 6.9) | 0.0 | 0.0 |
|  | Not married | NA | NA | NA | NA | NA | NA |
| **Europe & Central Asia** | | | | | | | |
| Albania (2017) | Married | 5.5 (1.8; 15.3) | 16.2 (1.8; 66.6) | 83.8 (33.4; 98.2) | 0.0 | 0.0 | 0.0 |
|  | Not married | 7.6 (2.0; 24.8) | 0.0 | 100 | 0.0 | 0.0 | 0.0 |
| Armenia (2015) | Married | 33.7 (12.4; 62.7) | 0.0 | 100 | 0.0 | 0.0 | 0.0 |
|  | Not married | 0.0 | 0.0 | 0.0 | 0.0 | 0.0 | 0.0 |
| Kyrgyzstan (2012) | Married | 29.8 (14.4; 51.7) | 66.1 (28.1; 90.6) | 33.9 (9.4; 71.9) | 0.0 | 0.0 | 0.0 |
|  | Not married | 58.0 (7.9; 95.7) | 0.0 | 100 | 0.0 | 0.0 | 0.0 |
| Tajikistan (2017) | Married | 8.1 (1.9; 28.3) | 66.2 (10.8; 96.9) | 33.8 (3.1; 89.2) | 0.0 | 0.0 | 0.0 |
|  | Not married | 0.0 | 0.0 | 0.0 | 0.0 | 0.0 | 0.0 |
| Turkey (2013) | Married | 29.7 (19.1; 43.1) | 56.5 (31.2; 78.9) | 43.5 (21.1; 68.9) | 0.0 | 0.0 | 0.0 |
|  | Not married | NA | NA | NA | NA | NA | NA |
| **South Asia** | | | | | | | |
| Afghanistan (2015) | Married | 18.4 (13.7; 24.4) | 36.5 (22.4; 53.4) | 63.5 (46.6; 77.6) | 0.0 | 0.0 | 0.0 |
|  | Not married | NA | NA | NA | NA | NA | NA |
| Bangladesh (2017) | Married | 67.9 (64.6; 71.0) | 24.8 (21.7; 28.3) | 70.1 (66.6; 73.5) | 3.4 (2.3; 5.0) | 1.5 (0.8; 2.7) | 0.1 (0.1; 0.1) |
|  | Not married | NA | NA | NA | NA | NA | NA |
| India (2015) | Married | 37.6 (36.0; 39.3) | 28.9 (26.0; 32.1) | 51.3 (48.2; 77.2) | 0.1 (0.0; 0.3) | 19.4 (17.2; 21.8) | 0.3 (0.1; 1.2) |
|  | Not married | 82.6 (67.1; 91.7) | 14.2 (6.5; 28.2) | 61.6 (43.1; 77.2) | 0 (0; 0) | 23.0 (10.2; 43.9) | 1.3 (0.4; 20.9) |
| Nepal (2016) | Married | 24.9 (20.0; 30.6) | 59.9 (47.1; 71.5) | 34.9 (23.1; 48.8) | 4.3 (1.4; 12.5) | 0.9 (0.1; 6.3) | 0.0 |
|  | Not married | 100.0 | 0.0 | 100 | 0.0 | 0.0 | 0.0 |
| Pakistan (2017) | Married | 23.3 (16.1; 32.6) | 33.3 (16.5; 55.8) | 59.6 (38.4; 77.7) | 0.0 | 7 (1.7; 25.3) | 0.0 |
|  | Not married | NA | NA | NA | NA | NA | NA |
| **East Asia & the Pacific** | | | | | | | |
| Cambodia (2014) | Married | 45.8 (37.2; 54.7) | 60.9 (47.2; 73.1) | 38.0 (25.8; 51.9) | 0.0 | 0.0 | 1.1 (1.1; 1.1) |
|  | Not married | 17.0 (3.6; 53.1) | 45.2 (4.9; 93) | 54.8 (7.0; 95.1) | 0.0 | 0.0 | 0.0 |
| Indonesia (2017) | Married | 81.7 (76.5; 86.0) | 30 (24.2; 36.5) | 70.0 (63.5; 75.8) | 0.0 | 0.0 | 0.0 |
|  | Not married | 12.9 (3.1; 40.9) | 0 | 100 | 0.0 | 0 | 0.0 |
| Myanmar (2015) | Married | 73.0 (64.9; 79.8) | 52.9 (42; 63.5) | 42.9 (32.5; 54.0) | 2.0 (0.5; 7.6) | 0 | 2.3 (2.3; 2.3) |
|  | Not married | 0.0 | 0.0 | 0.0 | 0.0 | 0 | 0 |
| Papua New Guinea (2016) | Married | 32.6 (22.3; 45.0) | 93.1 (73.8; 98.5) | 2.2 (0.3; 14.7) | 0.0 | 0 | 4.6 (4.6; 4.6) |
|  | Not married | 16.0 (8.3; 28.7) | 40.2 (13.2; 74.9) | 0.0 | 0.0 | 32.8 (8.6; 71.6) | 27.0 (26.7; 27.0) |
| Philippines (2017) | Married | 44.8 (37.3; 52.5) | 59.3 (47.2; 70.2) | 40.7 (29.8; 52.8) | 0.0 | 0.0 | 0.0 |
|  | Not married | 13.8 (5.7; 30.0) | 6.8 (0.8; 39.8) | 93.2 (60.2; 99.2) | 0.0 | 0.0 | 0.0 |
| Timor Leste (2016) | Married | 19.8 (11.6; 31.7) | 77.4 (42.6; 94.1) | 9.6 (1.4; 45.3) | 12.9 (1.9; 53.7) | 0.0 | 0.0 |
|  | Not married | 0.0 | 0.0 | 0.0 | 0.0 | 0.0 | 0.0 |
| **Latin America & the Caribbean** | | | | | | | |
| Colombia (2015) | Married | 71.8 (67.9; 75.5) | 47.0 (41.6; 52.6) | 47.1 (41.6; 52.8) | 3.7 (2.2; 6.2) | 0.0 | 2.1 (2.1; 2.1) |
|  | Not married | 77.9 (74.2; 81.2) | 17.3 (14.0; 21.2) | 75.8 (71.1; 80.0) | 4.5 (3.0; 6.9) | 0.0 | 2.3 (2.3; 2.3) |
| Dominican Republic (2013) | Married | 60.6 (53.0; 67.7) | 50.7 (41.9; 59.5) | 42.0 (32.8; 51.9) | 4.6 (1.8; 11.2) | 1.8 (0.4; 7.3) | 0.9 (0.8; 0.9) |
|  | Not married | 59.5 (49.2; 69.0) | 32.7 (20.5; 47.7) | 58.3 (43.3; 71.9) | 1.4 (0.3; 6.0) | 7.5 (2.7; 19.1) | 0.1 (0.1; 0.1) |
| Guatemala (2014) | Married | 50.1 (45.5; 54.8) | 67.8 (61.3; 73.6) | 26.6 (21.3; 32.8) | 3.5 (2.1; 5.9) | 1.1 (0.3; 4.2) | 1.0 (1.0; 1.0) |
|  | Not married | 48.1 (37.8; 58.6) | 7.5 (3.2; 16.5) | 78.8 (64.3; 88.4) | 10.5 (3.7; 26.2) | 3.3 (1.0; 10.2) | 0.0 |
| Haiti (2016) | Married | 28.7 (22.7; 35.6) | 45.9 (32.2; 60.1) | 21.0 (11.4; 35.5) | 31.2 (19.5; 45.8) | 2.0 (0.5; 8.2) | 0.0 |
|  | Not married | 27.3 (22.2; 33.0) | 27.8 (17.9; 40.5) | 43.9 (31.8; 56.7) | 1.3 (0.3; 5.6) | 27.1 (18.0; 38.6) | 0.0 |
| Honduras (2011) | Married | 67.4 (63.4; 71.1) | 54.9 (50; 59.7) | 35.0 (30.7; 39.6) | 7.5 (5.2; 10.9) | 1.3 (0.6; 2.8) | 1.2 (1.2; 1.2) |
|  | Not married | 59.6 (51.9; 66.9) | 20.4 (13.2; 30.2) | 72.5 (61.9; 81.0) | 2.2 (0.6; 8.3) | 3.4 (1.4; 8.2) | 1.5 (1.4; 1.5) |
| Peru (2020) | Married | 58.4 (50.8; 65.7) | 48.6 (41.3; 55.9) | 24.7 (17.5; 33.7) | 0.0 | 0.0 | 26.7 (26.6; 26.7) |
|  | Not married | 63.9 (49.5; 76.2) | 19.3 (11.5; 30.7) | 80.1 (68.4; 88.2) | 0.0 | 0.2 (0; 1.4) | 0.4 (0.4; 0.4) |
